# Phosphoproteomic mapping of CCR5 and ACKR2 signaling properties

**DOI:** 10.3389/fmolb.2022.1060555

**Published:** 2022-11-22

**Authors:** Alessandro Vacchini, Elisa Maffioli, Dario Di Silvestre, Cinzia Cancellieri, Samantha Milanesi, Simona Nonnis, Sabrina Badanai, Pierluigi Mauri, Armando Negri, Massimo Locati, Gabriella Tedeschi, Elena Monica Borroni

**Affiliations:** ^1^ IRCCS Humanitas Research Hospital, Milan, Italy; ^2^ Department of Medical Biotechnologies and Translational Medicine, University of Milan, Segrate, Italy; ^3^ Department of Veterinary Medicine and Animal Science, University of Milan, Lodi, Italy; ^4^ Institute of Technologies in Biomedicine, National Research Council (ITB-CNR), Milan, Italy; ^5^ CIMAINA, Milan, Italy

**Keywords:** ACKR2, CCR5, signaling, SILAC, phosphoproteome

## Abstract

ACKR2 is an atypical chemokine receptor which is structurally uncoupled from G proteins and is unable to activate signaling pathways used by conventional chemokine receptors to promote cell migration. Nonetheless, ACKR2 regulates inflammatory and immune responses by shaping chemokine gradients in tissues *via* scavenging inflammatory chemokines. To investigate the signaling pathways downstream to ACKR2, a quantitative SILAC-based phosphoproteomic analysis coupled with a systems biology approach with network analysis, was carried out on a HEK293 cell model expressing either ACKR2 or its conventional counterpart CCR5. The model was stimulated with the common agonist CCL3L1 for short (3 min) and long (30 min) durations. As expected, many of the identified proteins are known to participate in conventional signal transduction pathways and in the regulation of cytoskeleton dynamics. However, our analyses revealed unique phosphorylation and network signatures, suggesting roles for ACKR2 other than its scavenger activity. In conclusion, the mapping of phosphorylation events at a holistic level indicated that conventional and atypical chemokine receptors differ in signaling properties. This provides an unprecedented level of detail in chemokine receptor signaling and identifying potential targets for the regulation of ACKR2 and CCR5 function.

## Introduction

Atypical chemokine receptors (ACKRs) constitute a subgroup of four chemokine receptors, including ACKR1 (also known as Duffy Antigen Receptor for Chemokines), ACKR2 (also known as D6), ACKR3 (also known as CXCR7), and ACKR4 (also known as CCX-CKR) (Bachelerie, et al., 2014a; Bachelerie, et al., 2014b). Their role as regulatory elements of the chemokine network has been recognized in a wide range of developmental, physiological, and pathological contexts (Graham, et al., 2012; Cancellieri, et al., 2013a). In comparison to conventional chemokine receptors ACKRs do not directly induce leukocyte migration but rather, control their trafficking through the shaping of chemokine gradients within tissues. This function is supported by their unique trafficking properties, which allows the continuous uptake, transport, and/or presentation of their cognate ligands. The acknowledged “atypical” nature of ACKRs is based on their inability to support G protein-dependent signaling pathways as a consequence of structural alterations at different sites in their primary sequence, which in turn impairs their ability to couple to G proteins (Cancellieri, et al., 2013b). This has led to the hypothesis they use alternative signaling pathways, consistent with the increasing evidence that ACKRs require the β-arrestin pathway to shape chemokine gradients in tissues, suggesting that they represent a subgroup of β-arrestin-biased chemokine receptors (Vacchini, et al., 2016).

ACKR2 is a highly promiscuous receptor mainly expressed in trophoblasts, endothelial cells of afferent lymphatic vessels, and some leukocytic subsets (Cancellieri, et al., 2013a; Lee, et al., 2013). In these biological districts, ACKR2 is capable of binding, internalizing and scavenging the majority of inflammatory CC chemokines, a function that has been proven to be required for the appropriate resolution of inflammation, as well as the regulation of adaptive immune responses in several pathological conditions, including infections, allergies, and cancers (Bonecchi and Graham 2016). The ACKR2 scavenging function requires the β-arrestin1-dependent activation of the Rac1-PAK1-LIMK1-cofilin pathway, which finely regulates cytoskeletal dynamics and promotes both constitutive agonist-induced receptor internalization and recycling to the cell membrane (Bonecchi, et al., 2008; Borroni, et al., 2013a; Vacchini, et al., 2020). Thus, ACKR2 being structurally unable to couple G proteins, has adopted a β-arrestin-dependent pathway to control its trafficking and scavenging properties, in line with increasing evidence that β-arrestins may function as adaptor proteins for different signaling proteins (Shukla, et al., 2011; Gurevich and Gurevich 2013). Here we adopted a quantitative SILAC-based phosphoproteomic mapping approach to provide a comprehensive analysis of ACKR2 signaling properties in a tetracycline-inducible cell system. The agonist CCL3L1-induced activity of ACKR2 was characterized and compared with the signaling properties of conventional chemokine receptor CCR5, which also interacts and is activated by the CC inflammatory chemokine CCL3L1.

## Materials and methods

### Conditional expression of ACKR2 and CCR5

Conditional expression of CCR5 and ACKR2 was achieved using HEK293 T-Rex cells (Life Technologies), which stably express the tetracycline-responsive repressor protein and inhibit gene expression downstream to tetracycline-responsive operon. HEK293 T-Rex cells were maintained in complete D-MEM with 25 μg/ml blasticidin and were transfected using lipofection (Lipofectamine 2000, Invitrogen) with pcDNA4/Tet-on plasmids encoding HA-tagged ACKR2 and CCR5 under control of a tetracycline-responsive promoter. Cells were selected using 100 μg/ml zeocin (Life Technologies) and receptor expression was induced incubating cells with 1 μg/ml tetracycline. Analysis was performed 24 h after receptor expression induction.

### SILAC analysis

**FIGURE 1:**
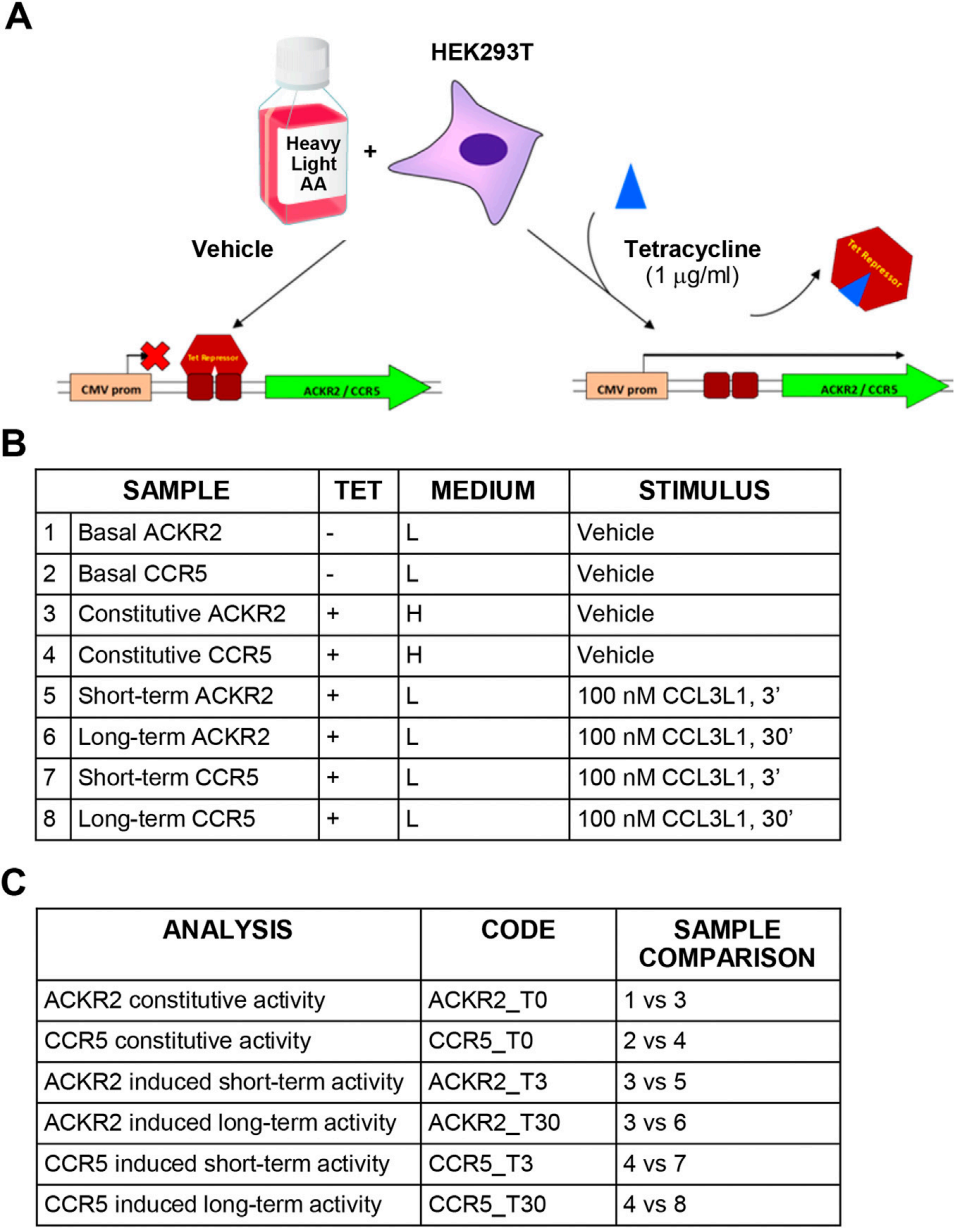
Experimental design and technical controls. **(A)** Schematic representation of the SILAC procedure. **(B)** Experimental samples generated for SILAC analysis. **(C)** Comparisons used to define constitutive and agonist-induced receptors’ activities.

SILAC D-MEM medium w/o glutamine, arginine and lysine (Life Technologies) was supplemented with 2 mM UltraGlutamine (Lonza) and either [^12^C_6_,^14^N_4_]-arginine/[^12^C_6_,^14^N_2_]-lysine or [^13^C_6_,^15^N_2_]-lysine/[^13^C_6_,^15^N_4_]-arginine (Cambridge Isotope Laboratories) to generate light or heavy mediums, respectively, with a final concentration of 21 mg/L for arginine and 48 mg/L for lysine. Complete medium was obtained adding 10% dyalized FBS (Life Technologies) and 100 U/ml pen-strep (Lonza) (complete SILAC D-MEM). During the adaptation phase, HEK293 T-Rex ACKR2 or CCR5 cells were grown in light or heavy SILAC D-MEM medium for five passages to achieve complete amino acid incorporation prior to further manipulation. Cells were then seeded onto plates for 24 h with either the presence or absence of 1 μg/ml tetracycline (Life Technologies) (Figure 1A). Complete SILAC D-MEM was then replaced with heavy or light SILAC D-MEM with 0.1% BSA, with or without tetracycline, and after 24 h 10^7^ cells were resuspended in 1 ml of heavy or light SILAC D-MEM with 0.1% BSA, incubated at 37°C with gentle shaking for 30 min and stimulated with 100 nM CCL3L1 or vehicle for the indicated time, then washed with ice-cold PBS and stored at −80°C. To investigate constitutive and agonist-induced signaling activities, samples for HEK293 T-Rex ACKR2 and CCR5 cells were generated and compared as reported in Figures 1B,C. Ten independent cell preparations of 10^7^ cells each were generated. Before SILAC analysis, each cell preparation was controlled for efficient tetracycline-dependent receptor induction by FACS analysis and for CCL3L1-dependent cofilin phosphorylation by Western blotting, as reported in Supplementary Figures S1A,B, respectively. To preserve sample quality and avoid loss of protein phosphorylation, cells were pelleted immediately after stimulation and stored at −80°C. Cell lysis was performed immediately before SILAC analysis in urea lysis buffer containing 20 mM HEPES pH 8.0, 8 M urea, 10 nM microcystin (Enzo Life Sciences), 10 nM calyculin A and phosphatases inhibitor cocktail (Cell Signaling). Cells were sonicated using Branson 250 Sonifier at 20% pulse for 5 s, for three times on ice. Samples were centrifuged at 15,000 × g for 10 min and protein concentration in the supernatant was measured using the Bradford method. Equal amounts of protein from heavy and light lysates were combined. Proteins were reduced with 13 mM DTT at 50°C for 15 min and cysteines were alkylated mixing with 26 mM iodoacetamide at room temperature in the dark for 30 min. The material was diluted to a final concentration of 2 M urea by the addition of 20 mM HEPES, pH 8.0, and digested overnight with sequencing-grade trypsin (Promega) at a 1:50 (enzyme:substrate) ratio in the presence of 1 mM methylamine. Digestion was quenched by addition of trifluoroacetic acid (TFA) to a final pH of 3. Precipitates were removed by centrifugation at 4,000 rpm for 30 min. Peptides were desalted on SepPak C18 columns (Waters) according to manufacturer’s instructions and eluted in 0.1% TFA/80% acetonitrile (ACN), divided in aliquots and subjected to hydrophilic interaction liquid chromatography (HILIC) using a 4.6 × 250-mm TSK gel Amide-80 5-μm particle column (Tosoh Biosciences) and a JASCO HPLC equipped with two PU-980 pumps and a Uvidec-100V detector (set at 220 nm) (Marinoni, et al., 2008). Peptides were loaded in 80% solvent B (100% ACN with 0.1% TFA). Solvent A consisted of 0.1% TFA in water. Peptides were eluted with a gradient consisting of 80% B held for 20 min followed by 80%–70% B for 10 min, 70%–60% B for 30 min, and 60%–0% B for 5 min. Fourteen fractions were collected throughout the gradient. Fractions from one aliquot were reduced to a very small volume using Savant Speed Vac concentrators (Thermo Fisher Scientific) to remove ACN, brought to 20 µL with 0.1% formic acid (FA), desalted using ZipTip (Sigma Aldrich) following the manufacturer’s instructions and submitted to mass spectrometric analysis. Fractions from other aliquots were pooled and further enriched in phosphorylated peptides using TiO_2_ beads (GL Science) as follows: the material was reduced to a small volume, brought to 200 µL with 300 mg/ml lactic acid in 80% acetonitrile:0.5% TFA and incubated for 3 h at room temperature with TiO_2_ beads activated following the manufacturer’s instructions (TiO_2_:sample 15:1 w/w) under agitation. Following centrifugation at 7,000 rpm for 3 min, the supernatant was discarded and the beads were washed 3 times through a 5 min incubation with 200 uL 80% ACN: 0.5% TFA, centrifuged at 7,000 rpm for 3 min; phosphopeptides were then eluted in two steps by addition of 200 uL 5% NH_4_OH, 30 min incubation at room temperature, 3 min centrifugation at 7,000 rpm, followed by the same protocol using 5% piperidine. Eluted materials were immediately brought to pH < 4 with FA and pooled. The volume was reduced using Savant Speed Vac concentrator and the mixture desalted with ZipTip and submitted to mass spectrometric analysis.

### LC-ESI MS/MS and post-acquisition analysis

Nano LC-ESI-MS/MS analysis was performed as previously reported (Zanotti, et al., 2016). The peptide mixture was separated online on a Dionex UltiMate 3000 HPLC System (Thermo Fisher Scientific) using a PicoFrit ProteoPrep C18 column (200 mm, internal diameter 75 μm) (New Objective). Gradient: 1% ACN in 0.1% FA for 10 min, 1–4% ACN in 0.1% FA for 6 min, 4–30% ACN in 0.1% FA for 147 min and 30–50% ACN in 0.1% FA for 3 min at a flow rate of 0.3 μL/min. The eluate was electrosprayed into an LTQ Orbitrap Velos (Thermo Fisher Scientific) through a Proxeon nanoelectrospray ion source (Thermo Fisher Scientific). The LTQ-Orbitrap was operated in positive mode in data-dependent acquisition mode to automatically alternate between a full scan (m/z 350–2000) in the Orbitrap (at resolution 60,000, AGC target 1000000) and subsequent CID MS/MS in the linear ion trap of the 20 most intense peaks from full scan (normalized collision energy of 35%, 10 ms activation). Isolation window: 3 Da, unassigned charge states: rejected, charge state 1: rejected, charge states 2+, 3+, 4+: not rejected; dynamic exclusion enabled (60 s, exclusion list size: 200). Multistage activation mode was enabled with neutral loss masses of 32.66, 48.99, and 97.97. Data acquisition was controlled through Xcalibur 2.0 and Tune 2.4 software (Thermo Fisher Scientific). Mass spectra were analyzed using MaxQuant software (version 1.3.0.5) (Schulte, et al., 2016). The initial maximum allowed mass deviation was set to 10 ppm for monoisotopic precursor ions and 0.5 Da for MS/MS peaks. Enzyme specificity was set to trypsin, defined as C-terminal to arginine and lysine excluding proline, and a maximum of two missed cleavages were allowed. Carbamidomethylcysteine was set as a fixed modification, phosphorylation of Ser, Thr, and Tyr, protein N-terminal acetylation, Met oxidation, Asn/Gln deamidation as variable modifications. The spectra were searched by the Andromeda search engine against the human UniProt sequence database (release 2014_01). Protein identification required at least one unique or razor peptide per protein group. The required false positive rate was set to 1% at the peptide, protein and site level, and the minimum required peptide length was set to 6 amino acids. The distribution of SILAC ratios was normalized within MaxQuant at the peptide level so that the median of log2 ratios is zero. Quantitative analyses were performed using the Perseus software (version 1.5.1.6). Only phosphopetides and proteins present and quantified in at least 2 out of 3 repeats were considered as positively identified in a sample (ACKR2_T0, ACKR2_T3, ACKR2_T30, CCR5_T0, CCR5_T3, and CCR5_T30) and used for further analyses. Geometric mean of biological replicate SILAC ratios were used to assess phosphorylation sites and protein relative quantification (Harsha, et al., 2008). Phosphorylation sites and proteins were considered up- or down- regulated if the geometric mean of SILAC ratio of replicates was >1.5 or <0.67, respectively (>50% change in level) (Kim, et al., 2013; Storvold, et al., 2013). The mass spectrometry proteomics data have been deposited to the ProteomeXchange Consortium *via* the PRIDE partner repository with the dataset identifier PXD009835, PXD009851, PXD009865, PXD009866, PXD009908, PXD00991.

### Network analysis

Two different *Homo sapiens* protein-protein interaction (PPI) network models were reconstructed using the STRING Cytoscape’s App starting from the differentially phosphorylated proteins (DPPs) characterized for CCR5 and AKCR2, respectively. Specifically, physical and/or functional interactions were filtered by considering only those “experiments” or/and “databases” annotated, with a STRING Score ≥0.15 and ≥0.35, respectively (Doncheva, et al., 2019). In addition, by the support of the GO enrichment tool inserted in STRING Cytoscape’s App, the DPPs were grouped in functional modules, while the most enriched pathways (by KEGG, Reactome and WikiPathways) were represented through a bar chart (FDR <0.05, *p* value < 0.001).

Starting from the same sets of DPPs in CCR5 and AKCR2, two further protein signaling network models were reconstructed by PesCa Cytoscape’s APP (Scardoni, et al., 2015) by taking into consideration activation, inhibition and docking protein relationships. The reconstructed networks were analyzed at topological level by CentiScaPe Cytoscape’s App (Scardoni, et al., 2014); all centralities (Betweenness, Bridging, Centroid, Closeness, Eccentricity, EigenVector, Radiality, Stress, InDegree and OutDegree) were calculated, and the hub proteins were selected by Betweenness as previously reported (Sereni, et al., 2019); specifically, only proteins with a mean value of Betweenness above the mean were considered hubs. Statistical significance of all topological results was tested by considering randomized network models; they were reconstructed and analyzed by an *in-house* R script based on VertexSort (to build random models), igraph (to compute centralities), and ggplot2 (to plot results) libraries; results were visualized in the form of Violin plots.

### GRK2 knockdown

Silencing of GRK2 expression was achieved by siRNA technology. Lipofectamine RNAiMAX (Thermo Fisher Scientific) was used to transfect HEK293 T-Rex cells with 50 nM ON-TARGETplus siRNAs against GRK2 or scrambled control, according to manufacturer’s instructions (Dharmacon). After 24 h, receptor expression was induced with 1 μg/ml tetracycline for further 24–48 h.

### Protein analysis

To assess protein expression and phosphorylation, Western blotting and WES technology were employed, respectively.

Western blotting was performed as previously described (Borroni et al., 2013b). In brief, 20–50 µg total proteins were used to run SDS-PAGE and transferred to polyvinylidene fluoride (PVDF) membranes (Bio-Rad) that were incubated at 4°C O/N under constant shaking with primary antibodies against GRK2 (Santa Cruz Biotechnology) and anti-HA.11 Epitope Tag antibody (clone 16B12, Covance). To reveal the primary antibodies, horseradish peroxidase-conjugated antibodies were used (all from GE Healthcare). HRP substrate Immobilon Western (Millipore) was used to acquire blot images on ChemiDoc XRS Imaging System (Bio-Rad). Densitometric analysis was performed by Quantity One software (Bio-Rad) and protein band intensity was calculated by normalization over α-tubulin band intensity.

WES System (Protein Simple, Biotechne) was performed according to the manufacturer’s instructions and using the Compass software. Briefly, the following reagents were used: EZ Standard Pack (Protein Simple PS-ST01EZ-8), Anti-Rabbit Detection Module (Protein Simple DM-001), 12–230 kDa Wes Separation Module (Protein Simple W004-1). In total, 0.4 μg/μL of protein sample was loaded. The voltage used was 375 V for a separation time of 25 min. The incubation time used for the primary and secondary antibodies was 30 min each. The following primary antibodies from Cell Signaling Technologies were used: Phospho-p44/42 MAPK (Erk1/2) (Thr202/Tyr204) #9101, p44/42 MAPK (Erk1/2) Antibody #9102, Phospho-Akt (Ser473) #9271 and Akt Antibody #9272.

### Chemokine scavenging assay

ACKR2 and CCR5-expressing CHO-K1 cells (5 × 10^4^) were incubated in 10% FCS medium at 37°C for 18 h in 96-wells plates previously coated for 30 min at 37°C with 100 µL/well poly-lysine. Cells were pretreated 1 h with 10 µM UO126 and Triciribine (Calbiochem) and further incubated at 37°C in culture media + 1% BSA + 25 mM HEPES supplemented with 1 nM CCL3L1 for the indicated time points. Chemokine concentration in the supernatant was measured by ELISA, according to manufacturer’s instruction (R&D Systems).

## Results

### ACKR2 and CCR5 triggers distinct phosphorylation events after agonist activation

**FIGURE 2:**
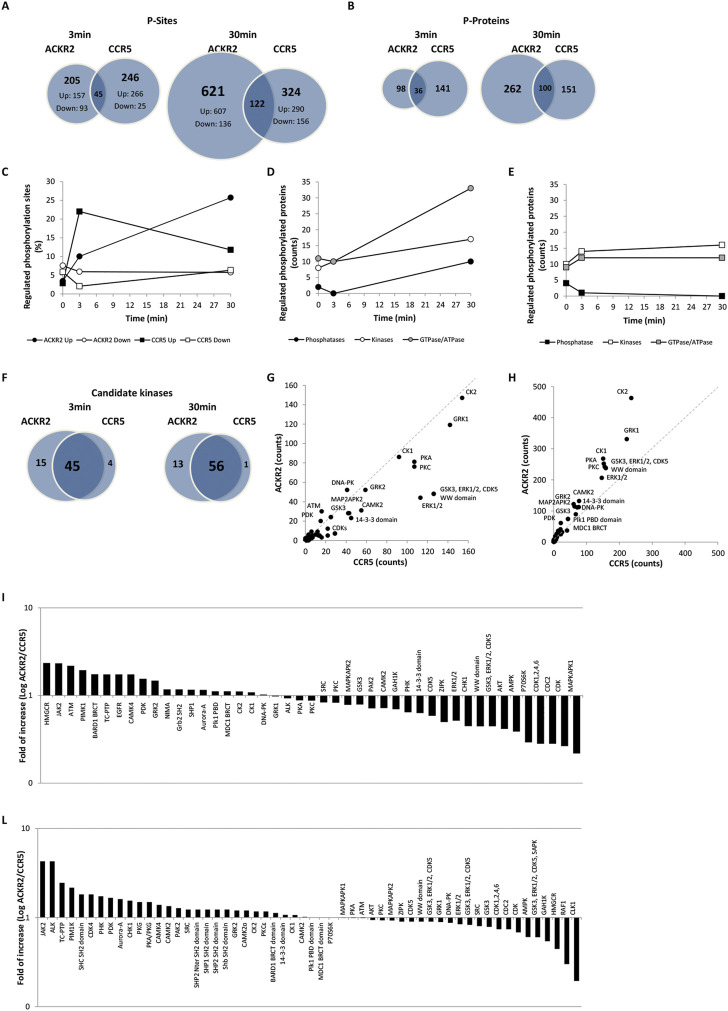
CCL3L1-induced phosphoproteome in ACKR2- and CCR5-expressing cells. **(A–B)** Number of distinct and shared phosphosites **(A)** and phosphoproteins **(B)** after ACKR2 and CCR5 short-term (T3) or extended activation (T30) activation with 100 nM CCL3L1. **(C)** Kinetics of up (black) and down (white) regulated phosphosites after ACKR2 (circle) and CCR5 (square) activation with 100 nM CCL3L1. **(D–E)** Kinetics of up and downregulated phosphoproteins after ACKR2 **(D)** and CCR5 **(E)** activation with 100 nM CCL3L1, distinct for kinases (white), phosphatases (black), and GTPase/ATPases activity (grey). **(F–H)** Candidate kinases **(F)** identified by sequence motif analysis in ACKR2 and CCR5 regulated phosphosites are shown as absolute counts of related phosphorylation sequence motifs at T3 **(G)** and T30 **(H)** time points. **(I–L)** ACKR2/CCR5 ratio of the percentage of phosphorylation sequence motif counts attributed to candidate kinases active at T3 **(I)** and T30 **(L)** time points after stimulation with 100 nM CCL3L1.

To investigate the signaling properties of conventional and ACKRs, we carried out a comparative analysis of phosphosites detected in cells expressing the prototypic conventional chemokine receptor CCR5 or the related atypical chemokine receptor ACKR2 after short- and long-term activation by CCL3L1, which acts as an agonist at both receptors. Experiments were based on SILAC mass spectrometry quantitative phosphoproteomic analysis, with the main results reported in Supplementary Dataset S1. Overall, the SILAC-based phosphoproteomic mapping approach applied quantified 11,463 phosphosites in 5,090 phosphoproteins, which include 409 phosphoproteins uniquely regulated by CCR5 and 473 phosphoproteins uniquely regulated by ACKR2, most of which have not been previously implicated in chemokine receptor signaling. The effect of agonist induced ACKR2 and CCR5 signaling activity on the cell phosphoproteome was analyzed after stimulation with a common agonist CCL3L1 (100 nM) at short (3 min; T3) and prolonged (30 min; T30) time points. With the short time point, ACKR2 activation resulted in the modulation of 250 phosphosites in 134 phosphoproteins, which increased to 743 phosphosites in 362 phosphoproteins at T30. Under the same experimental conditions, CCR5 activation had effects comparable to ACKR2 at short time point, with 291 phosphosites in 177 phosphoproteins at T3, but was significantly less effective at later time point, with 446 phosphosites in 251 phosphoproteins at T30 (Figures 2A,B, Supplementary Table S1A and Supplementary Dataset S1). For both receptors, upregulated phosphosites were more abundant than downregulated ones at any time point, with peak values reached at short (T3) and late (T30) time points for CCR5 and ACKR2, respectively (Figure 2C and Supplementary Table S1A). Of note, only a minor fraction of phosphosites and target proteins were regulated in a coherent way by the two receptors, suggesting that ACKR2 and CCR5 signaling activity results in largely distinct effects (Figures 2A,B). Similar results were observed in a comparative analysis on the effects of ACKR2 and CCR5 expression on the cell phosphoproteome (Supplementary Figure S2A,B; Supplementary Table S2A and Supplementary Dataset S1) and proteome (Supplementary Figure S2F,G, Supplementary Table S3, and Supplementary Dataset S2) in the absence of the agonist, confirming that both conventional and ACKRs are endowed with a constitutive but profoundly different signaling activity (Gilliland, et al., 2013; Vacchini, et al., 2016).

The largely distinct phosphoproteomic events induced by ACKR2 and CCR5 suggests that they recruit and activate different proximal effectors. This hypothesis was supported by the kinetics of phosphorylation events occurring in different families of signal transducers, as kinases and GTP/ATPases showed kinetics profiles similar for the two receptors, while phosphatases were significantly regulated by ACKR2 activation at late time point but unaffected by CCR5 activation (Figures 2D,E and Supplementary Table S1B,C). Taken together, these results suggest that kinase activation is predominant compared to phosphatase activity but occurs with different kinetics between the two receptors, and that ACKR2 may trigger an extended signaling activity supported by GTP/ATPases.

Kinases that were potentially responsible for agonist-induced phosphorylation events were predicted based on an analysis of the sequence motifs from regulated phosphosites. Even if the majority of kinases were predicted to be activated by both receptors (Figure 2F and Supplementary Table S1D,E), their relative efficacy showed a time-related difference between CCR5- and ACKR2-activated cells. At the short time point there were 26 kinases more active with CCR5 compared to 19 with ACKR2 (Figures 2G,I and Supplementary Table S1D,E). At the extended time point there were 30 kinases more active with ACKR2 versus 25 with CCR5 (Figures 2H,L, and Supplementary Table S1D,E). Similar results were observed in absence of the agonist (Supplementary Figures S2C–E and Supplementary Table S2B) indicating that, although these receptors largely operate through a common set of kinases, some of these kinases are more relevant for one than the other and possibly orchestrate distinct effects.

### Functional implications of agonist induced ACKR2 and CCR5 signaling activity

To gain insights into the functional relevance of the agonist-activated signaling activity of the two receptors a PPI and signaling network analysis was performed. This revealed the prevalence of regulated protein phosphorylation within several known pathways involved with chemokine signaling, but also highlighted some groups of proteins that are not usually associated with chemokines.

**FIGURE 3:**
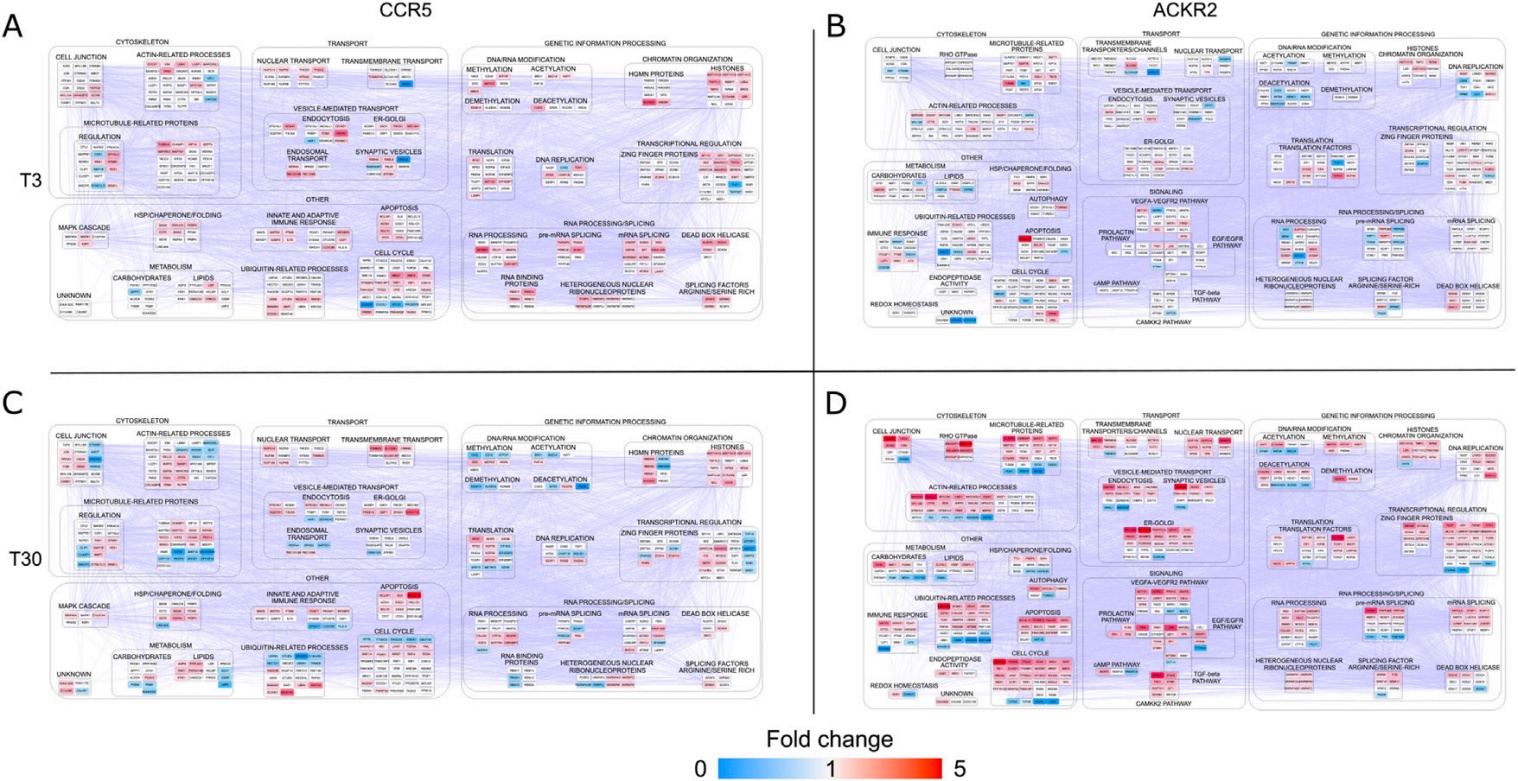
Protein-protein interaction (PPI) network models reconstructed starting from differentially phosphorylated proteins (DPPs) in CCR5 and ACKR2. Functional modules enriched in HEK-293T cells expressing CCR5 **(A)** and **(C)** and ACKR2 **(B)** and **(D)** after short-term [3’; T3 **(A)** and **(B)**] or extended [30’; T30 **(C)** and **(D)**] activation with 100 nM CCL3L1. Network models (CCR5: 435 nodes and 4,911 edges; ACKR2: 490 nodes and 7,151 edges) were reconstructed by STRING Cytoscape’s APP. Node color agrees with the fold change of phosphorylated proteins at T3 and T30.

At the short time point (T3), an initial effect of ACKR2 and CCR5 activation was the reinforcement of their influence on biological pathways that are also affected by their constitutive signaling properties (Supplementary Figure S3). CCR5 stimulation had impact on events classically associated to endocytosis, cytoskeletal organization, and ubiquitination, as well as on several unexpected nuclear-related functions. This included RNA metabolism, chromatin organization/transcriptional regulation and DNA metabolism, thus having potential implications on the cell cycle and cell proliferation (Figure 3A). These results are consistent with the recently reported proteomic data that were observed after activation of other conventional chemokine receptors such as CCR2 (Huang, et al., 2020). ACKR2 stimulation on the other hand, had an impact on major functional events (Figure 3B). Although the majority were shared with CCR5, a large fraction was specific for ACKR2.

A limited number of functional categories were affected by the prolonged triggering of CCR5 (T30), which did not elicit major functional events in addition to those already detected at the short time point (Figure 3C). Conversely, a significant modulation of these functional categories was observed after prolonged ACKR2 triggering (Figure 3D), with several functional consequences observed, including an influence on cytoskeletal components, consistent with previously reported data (Borroni et al., 2013a; Vacchini, et al., 2020). Notably, these findings are in concordance with the pathways that were most enriched, taking into consideration the Reactome, WikiPathways and KEGG databases (Supplementary Figure S4 and Supplementary Table S4). Although the majority were shared between ACKR2 and CCR5, a large fraction were specifically enriched for the ACKR2 dataset. Specifically, they included processes involved in signaling by RHO GTPase, membrane trafficking and chromatin modification (by Reactome). They also involved a group of signaling pathways, which included VEGFA-VEGFR, TGF-β, CAMKK2 and prolactin signaling (by WikiPathways), and EGF/EGFR and insulin signaling (by KEGG).

### Signaling network hubs of ACKR2 and CCR5

**FIGURE 4:**
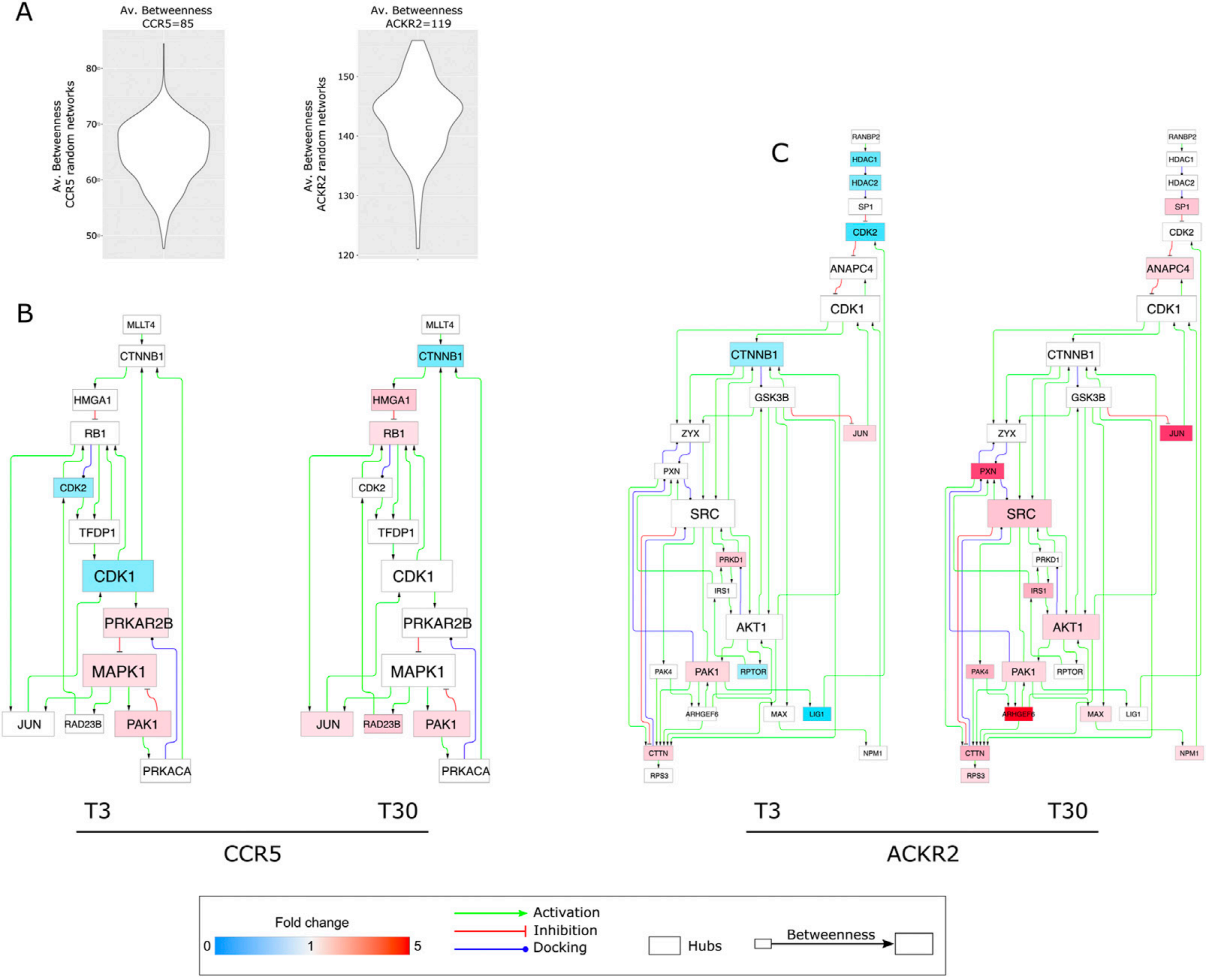
Protein hubs characterizing CCR5 and ACKR2 signaling network models. **(A)** Violin plots reporting the average betweenness values from CCR5 and ACKR2 signaling random network models. **(B)** Protein hubs selected by betweenness in CCR5 signaling network (80 nodes and 144 edges). **(C)** Protein hubs selected by betweenness in ACKR2 signaling network (135 nodes and 276 edges). Node color agrees with the fold change of phosphorylated proteins at T3 and T30, while node and font size are in agreement with the corresponding betweenness value.

With the intent of exploring the resulting enriched processes in further depth, we analyzed two signaling network models reconstructed from the DPPs in CCR5 and ACKR2. Following their topological evaluation, we extracted a set of hub proteins which represented the most relevant molecules in the coordination of signaling pathways in CCR5 and ACKR2. For both receptors, the baseline effects were evident as a consequence of their constitutive signaling (Supplementary Figure S5), but these were dramatically enhanced after agonist-dependent triggering, at short and extended times for CCR5 and ACKR2, respectively (Figure 4 and Supplementary Table S5). MAPK1, CDK1, and PRKAR2B were the highest ranked hubs (by betweenness) in CCR5, while Src, CDK1, and AKT1 were the highest ranked hubs in ACKR2. Of note, in addition to CDK1, hubs in both CCR5 and ACKR2 signaling included CDK2, CTNNB1, JUN, and PAK1.

In ACKR2, hubs were mainly observed after prolonged receptor triggering, with several functional consequences including the influence on cytoskeletal components (i.e., PAK1) that are consistent with previously reported data (Borroni et al., 2013b). Notably, the identification of Src as the highest ranked hub in ACKR2 may explain the significant enrichment of phosphosites associated to G Protein-Coupled Receptor Kinase 2 (GRK2). Also known as Beta-Adrenergic Receptor Kinase 1 (βARK1) (Figure 2I–L and Supplementary Figure S2E) GRK2 is known to orchestrate chemokine receptors desensitization *via* the recruitment of β-arrestins (Reiter and Lefkowitz 2006), which in turn regulates GRK2 levels and the phosphorylation of endocytic proteins (i.e., dynamin I) involved in clathrin-dependent internalization and desensitization of GPCRs *via* Src-dependent activity (Luttrell and Miller 2013; Penela 2016). Additionally, it has been demonstrated that β-arrestins control ACKR2 stability (McCulloch, et al., 2008) and intracellular distribution. (Borroni, et al. 2013a). Consistent with this we observed that GRK2 expression was needed to protect ACKR2 from degradation (Supplementary Figure S2F).

### Biological relevance of hubs activation by ACKR2

**FIGURE 5:**
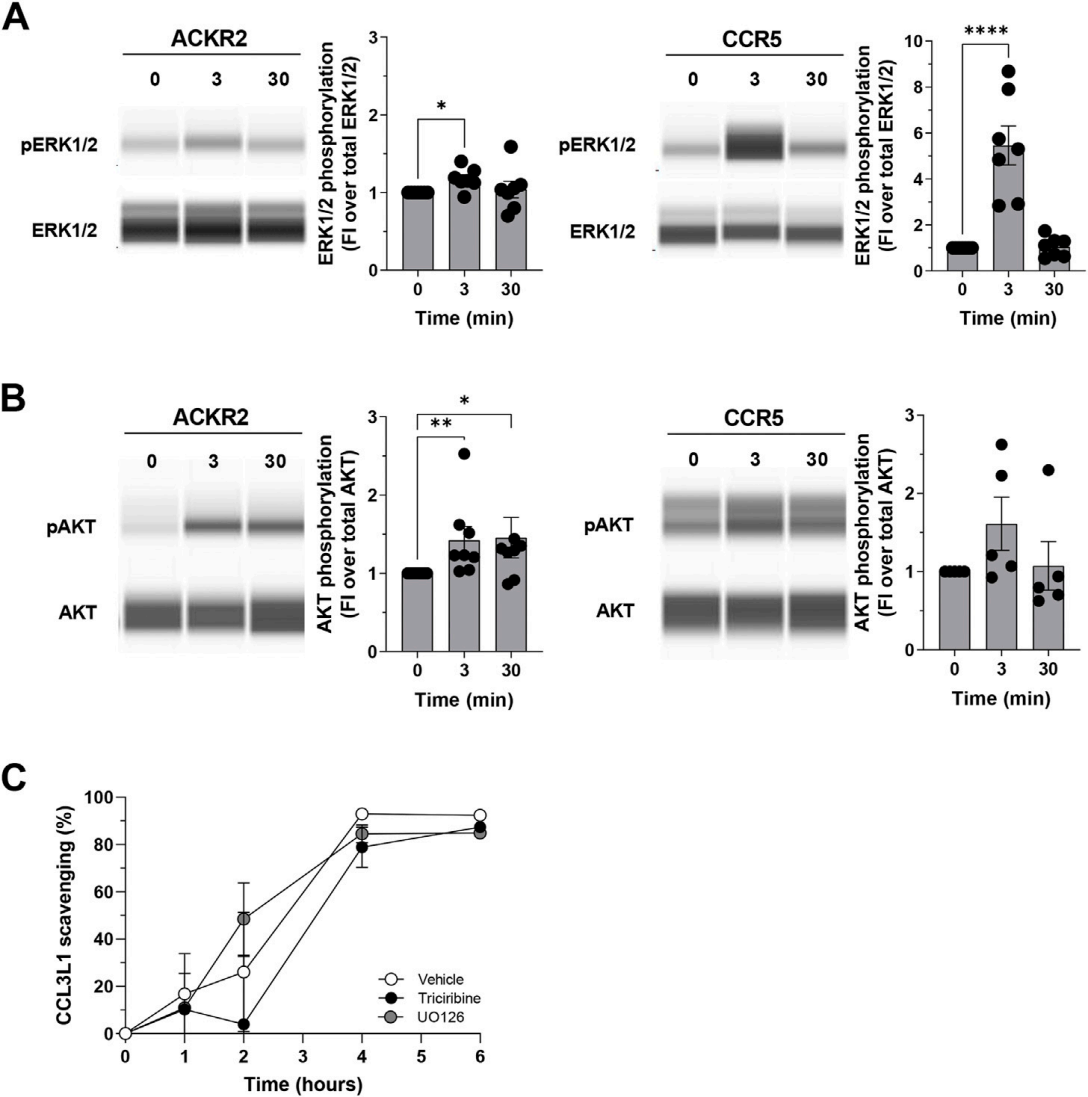
Biological relevance of ERK1/2 and AKT in ACKR2 and CCR5 cells. **(A–B)** ERK1/2 phosphorylation at T202/Y204 residue **(A)** and AKT phosphorylation at S473 residue **(B)** in ACKR2 and CCR5-expressing HEK293 T-Rex cells following stimulation with 100 nM CCL3L1 at T3 and T30 time points. Representative blots are shown on the left, quantification of the relative number of phosphorylated proteins is shown on the right and is indicated as fold increase in phosphorylated proteins abundance compared to that in untreated cells. Data information: statistical analysis by Kruskal–Wallis or ordinary One-Way ANOVA’s multiple comparison test (*p < 0.05, **p < 0.01, ***p < 0.005 for CCL3L1-stimulated cells versus untreated cells). FI: Fold of increase. **(C)** CCL3L1 degradation rate in ACKR2-expressing CHO-K1 cells stimulated with 100 nM CCL3L1 in presence or absence of the ERK1/2 inhibitor UO126 (10 µM) and the AKT inhibitor Triciribine (10 µM). The amount of intact chemokine present in the cell supernatant at indicated time points was quantified by ELISA. DMSO was used as vehicle for both inhibitors. Data information: results are the mean ± SEM of *n* = 2 independent experiments.

As previously reported, the functional analyses of phosphoproteasomes led to the prediction of several hubs orchestrating ACKR2 and CCR5 signaling architecture, which were more evident at the short time point for CCR5, and at extended time point for ACKR2. As expected, our set of hubs is enriched for proteins involved in several signal transduction pathways known to be activated upon chemokine receptor stimulation and are consistent with the recently reported proteomic data observed after triggering of CCR2 (Huang, et al., 2020). As a first step towards the validation of our observations, Western blotting was used to quantify the phosphorylation of several proteins of interest. Interestingly, phosphorylation of PAK1 has already been observed in a previous publication (Borroni et al., 2013a). One of the hallmarks of chemokine activation is the phosphorylation of MAPK/ERK. Data confirmed ERK1/2 activation with similar kinetics in both ACKR2 and CCR5-activated cells (Figure 5A and Supplementary Figure S6A), peaking at T3 after stimulation and then returning to baseline levels at T30. On the contrary, prolonged AKT1 activation was observed in ACKR2-activated cells, but not significatively confirmed in CCR5-activated cells (Figure 5B, Supplementary Figure S5B). Notably, these findings were confirmed in CHO cells that were stably expressing ACKR2 (Supplementary Figure S6C), indicating that the receptor retains intrinsic signaling properties regardless cellular background.

The above reported results indicate that several kinases may influence ACKR2 signaling properties, suggesting that ACKR2 signaling may impact on some ERK1/2 and AKT-related biological functions. As ACKR2 was able to activate these signaling pathways upon agonist engagement, and as ACKR2 trafficking supports receptor scavenging activity in an agonist-dependent manner, we first hypothesized a role for ERK1/2 and AKT on the chemokine scavenging activity of ACKR2, which at present represents its main biological function (Bonecchi, et al., 2008). However, when the efficacy of ACKR2-mediated CCL3L1 scavenging was investigated, ERK1/2 and AKT inhibition by UO126 or Triciribine treatment had no detectable effect (Figure 5C), indicating that the ACKR2-mediated activation of these two kinases was not relevant for its scavenger function, unlike PAK1 (Borroni, et al. 2013b).

## Discussion

In this study we report a comparative profiling of phosphoproteome events downstream to chemokine receptor CCR5 and its atypical counterpart ACKR2, both investigated short (3 min) and long-term (30 min) stimulation with the common agonist CCL3L1, an inflammatory CC chemokine. Results show that these two receptors, which may be considered representative examples of conventional and atypical chemokine receptors, being highly related from the structural point of view and with shared ligands, are characterized by significant differences in agonist-dependent signaling properties.

The detailed analysis of agonist-induced phosphoproteins regulated by both ACKR2 and CCR5, after short and prolonged stimulation with CCL3L1 reveal that the impact on the cell phosphoproteome was faster for CCR5 compared to ACKR2, suggesting significant differences in the recruitment and activation of proximal signal transducers. This hypothesis is consistent with the role of β-arrestins as GPCR signal adaptor proteins involved in the activation of a second wave of G proteins-independent signaling events (Jean-Charles, et al., 2017). G proteins and β-arrestins signaling modules rely on signals that are known to be partly shared but temporally and spatially diverse (Sposini and Hanyaloglu 2017). Furthermore, the analysis shows that, upon agonist engagement ACKR2 and CCR5 resulted in a similar number of regulated phosphosites and target proteins, but only a minor fraction of them were commonly regulated by the two receptors, suggesting that their signaling activities have largely distinct effects. In accordance, although both ACKR2 and CCR5 share the same fingerprint of predicted kinases, some of these are more relevant for one than the other, indicating that each receptor operates on a distinct set of signaling proteins. This is consistent with data on extracted hub proteins showing a distinct profile of molecules recruited by ACKR2 and CCR5, in coordinating their downstream signaling pathways. Similar results were observed in the comparative analysis of the effects of ACKR2 and CCR5 expression on the cell phosphoproteome and proteome in the absence of the agonist. Constitutive signaling has been reported for both conventional and atypical chemokine receptors (Hall 2000; Wan, et al., 2002; Slack and Hall 2012; Gilliland, et al., 2013; Vacchini, et al., 2016), and furthermore, mutations enabling chemokine-independent receptor signaling properties have also been reported (Alvarez Arias, et al., 2003). However, a comprehensive analysis of the signaling pathways activated by chemokine receptors’ constitutive activity is missing and their functional relevance in ACKR2 and CCR5 will require further investigation in order to exclude tetracycline-dependent phosphorylation events from our datasets, given the results on the modulatory effect on several intracellular signaling pathways of tetracycline and its analog reported in (PMID: 29124230).

Gene enrichment analysis showed that both ACKR2 and CCR5 stimulation resulted in the reinforcement of their influence on the biological pathways which are also affected by their constitutive activation. When ACKR2 is considered, a prominent gene signature is represented by cytoskeleton and transport regulation. Actin dynamics are essential for the chemotactic activity of chemokines (Barreiro, et al., 2007), with conventional chemokine receptors coordinating the acquisition of a migratory phenotype in leukocytes through the synchronous activation of both G proteins and β-arrestin signaling modules, which promote actin reorganization and activate small GTPases and cofilin phosphorylation (McGovern and DeFea 2014). Although G protein activation and migratory activity has never been reported for ACKR2, these data are consistent with our previous reports of a β-arrestin1-dependent Rac1-PAK1-LIMK1-cofilin signaling pathway promoting a massive rearrangement of actin filaments, essential for ACKR2 vesicular transport and receptor-mediated chemokine-scavenging activity (Borroni et al., 2013a). Gene enrichment analysis also highlighted GRK2 as a main effector downstream to ACKR2, with results demonstrating that GRK2 controls ACKR2 stability. This finding is in agreement with previous reports which indicate an involvement of β-arrestins in ACKR2 stability (McCulloch et al. 2008, Vacchini et al. 2020), as the GRK2/Gβγ-mediated phosphorylation of GPCRs has been shown to be mandatory for β-arrestins engagement (Dwivedi et al. 2018). Recent evidence indicates that the GRK2-mediated recruitment of β-arrestins may also occur independently of Gβγ signaling (Pack et al. 2018), and it is finely tuned by a β-arrestin-dependent Src activity (Penela 2016). This is in line with evidence that Gβγ activation by ACKR2 is not been detectable (Borroni et al. 2013a) and Src emerged as the highest ranked hub for ACKR2 in our dataset, strengthening the role of β-arrestins in ACKR2 signaling.

Aside from roles involving the cytoskeleton and transport, gene enrichment analysis revealed other prominent gene signatures, including several signaling pathways downstream to ACKR2 that are in line with evidence reported in the literature. The relationship with VEGFA-VEGFR signaling is consistent with previously reported observations on ACKR2 expression in lung endothelial cells (Hansell et al., 2020). Furthermore, TGFβ signaling, which has known influences on ACKR2 expression (McKimmie, et al. 2013) plays a role in skin fibrosis (Butenko, Ben Jashar et al., 2021). The reported role of ACKR2 in the systemic regulation of glucose tolerance, accompanied by reduced insulin secretion and increased whole body insulin sensitivity (Zheng, et al. 2016, Fioravante et al. 2019) is consistent with the identified enrichment in the insulin signaling pathway. Moreover, the novel role for ACKR2 in ductal epithelial branching required for the postnatal development of the mammary gland (Wilson, et al. 2017, Wilson, et al. 2020) is consistent with the prolactin signaling that is known to be essential for the branching phenotype of the mammary gland (Slepicka et al. 2021). Interestingly, the identified EGF-EGFR signaling has also been involved in mammary development and has been also described for ACKR3 (Salazar, et al. 2014). In addition to the above effects, gene enrichment in several pathways related to genetic information processing and cell cycle was observed for both receptors, consistent with recent data reported for CCR2 whose phosphoproteomic profile revealed numerous proteins that function in the nucleus (Huang, et al. 2020). This is not unexpected, considering that chemokine stimulation is known to up-regulate transcription and protein synthesis, as well as cell proliferation (O'Hayre, et al. 2008). However, the identification of multiple proteins that were not previously known to be regulated by chemokines, highlights the level of detail provided by the phosphoproteomics approach and its potential to yield novel information.

ACKR2 is a “professional” scavenger receptor involved in the resolution of chemokine-driven inflammatory responses through efficient degradation of inflammatory chemokines (Bonecchi et al., 2004). The identification of the ERK and AKT pathways downstream to ACKR2 and evidence that these signaling are not involved in chemokine scavenging also suggest that this atypical receptor still retains unknown biological properties. Moreover, in the case of both kinases, G protein- and β-arrestin-dependent activation occurs and is consistent with the β-arrestin1-biased signaling activity of ACKR2 (Bonecchi et al., 2013a) and the balanced signaling activity of CCR5 (Oppermann 2004). Interestingly, β-arrestin-dependent ERK and AKT activation has also been reported for ACKR3, a second “professional” chemokine scavenger receptor (Rajagopal et al., 2010, Torossian et al., 2014). For ACKR3, ERK and AKT activation has been shown to be essential in promoting cancer cell survival, proliferation and tumor angiogenesis, whilst the biological relevance for ACKR2 is still unknown. A scavenging-independent activity has been recently reported for ACKR2 when expressed on apoptotic neutrophils, where ACKR2 was instrumental for the resolution of inflammation through promoting efficient efferocytosis and shifting macrophages towards a pro-resolving phenotype (Pashover-Schallinger et al., 2012, Aswad et al., 2017). Of note, gene enrichment in apoptotic signaling has been reported in ACKR2, with BCL2L13, a BCL2-like protein (Meng et al., 2021), as the highest regulated phosphoprotein identified after agonist stimulation.

To conclude, this study represents the first extensive investigation of the phosphoproteome downstream to conventional and atypical chemokine receptors, and expands our current knowledge about ACKRs, providing the molecular background to advance studies on the biological processes involved in chemokine activities.

## Data Availability

The datasets presented in this study can be found in online repositories. The names of the repository/repositories and accession number(s) can be found below: http://proteomecentral.proteomexchange.org/cgi/GetDataset PXD009835, PXD009851, PXD009865, PXD009866, PXD009908, PXD00991.
