# Supplementary material for: Phosphoproteomic mapping of CCR5 and ACKR2 signaling properties
Source: Front Mol Biosci. 2022 Nov 22;9:1060555. doi: 10.3389/fmolb.2022.1060555 (PMC9723398; doi:10.3389/fmolb.2022.1060555)

A

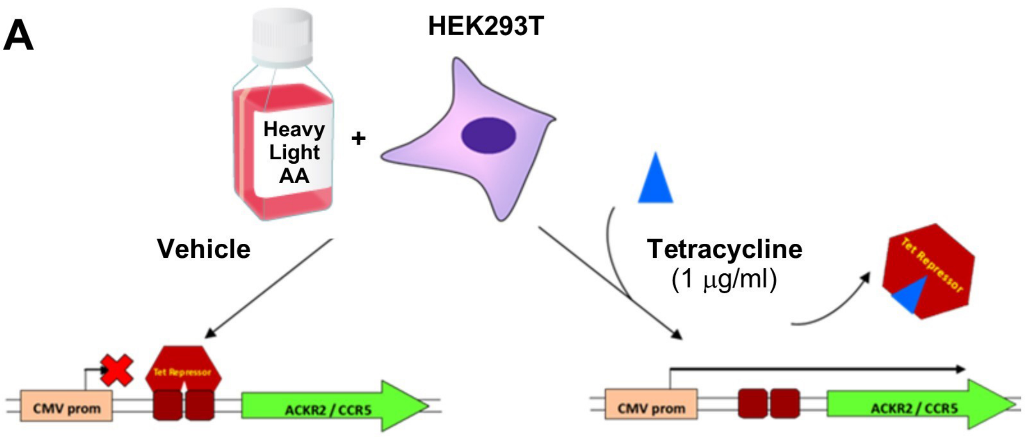

B

	SAMPLE	TET	MEDIUM	STIMULUS
1	Basal (ACKR2 cells)	-	Light	Vehicle
2	Basal (CCR5 cells)	-	Light	Vehicle
3	ACKR2 constitutive	+	Heavy	Vehicle
4	CCR5 constitutive	+	Heavy	Vehicle
5	ACKR2 short-term	+	Light	100 nM CCL3L1, 3’
6	ACKR2 long-term	+	Light	100 nM CCL3L1, 30’
7	CCR5 short-term	+	Light	100 nM CCL3L1, 3’
8	CCR5 long-term	+	Light	100 nM CCL3L1, 30’

C

ANALYSIS	CODE	SAMPLE COMPARISON
ACKR2 constitutive activity	ACKR2_T0	1 vs 3
CCR5 constitutive activity	CCR5_T0	2 vs 4
ACKR2 induced, short-term activity	ACKR2_T3	3 vs 5
ACKR2 induced, long-term activity	ACKR2_T30	3 vs 6
CCR5 induced, short-term activity	CCR5_T3	4 vs 7
CCR5, induced long-term activity	CCR5_T30	4 vs 8

D

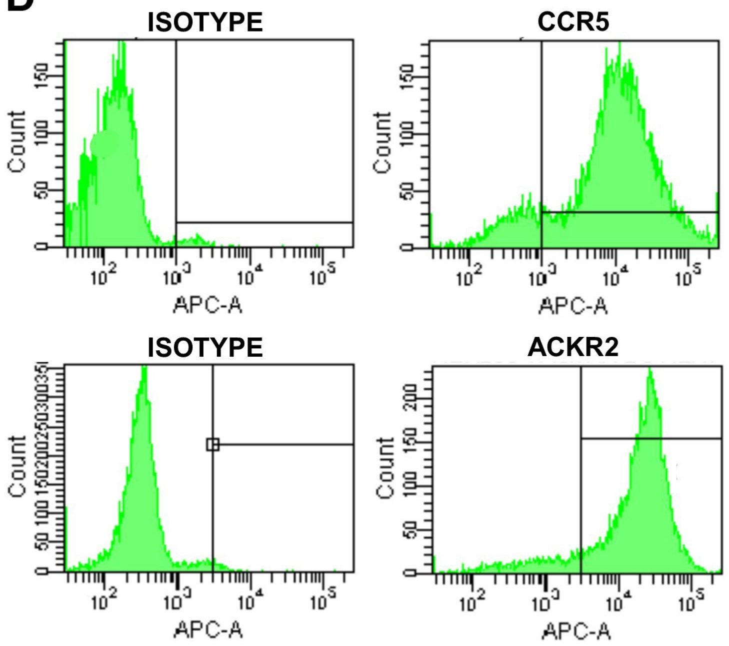

E

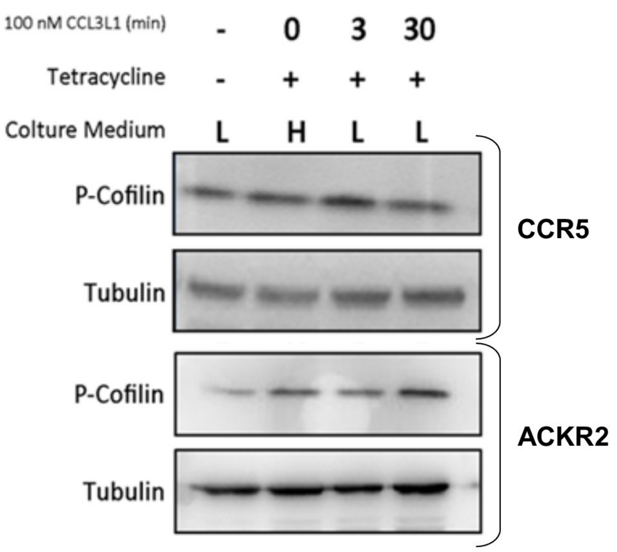

Supplement: Supplementary file 1 [file DataSheet1.zip › Data Sheet ZIP folder/Figure S1.pdf]
