# Supplementary material for: Phosphoproteomic mapping of CCR5 and ACKR2 signaling properties
Source: Front Mol Biosci. 2022 Nov 22;9:1060555. doi: 10.3389/fmolb.2022.1060555 (PMC9723398; doi:10.3389/fmolb.2022.1060555)
Supplement: Supplementary file 1 [file DataSheet1.zip › Data Sheet ZIP folder/Supplementary Material.pdf]

Supplementary Material

SUPPLEMENTARY DATASETS

Dataset S1.

Non-redundant phosphopeptide detection and quantification data obtained from each individual experiment.

Dataset S2.

Non-redundant protein detection and quantification data obtained from experiments following constitutive expression of ACKR2 and CCR5.

SUPPLEMENTARY FIGURES

A

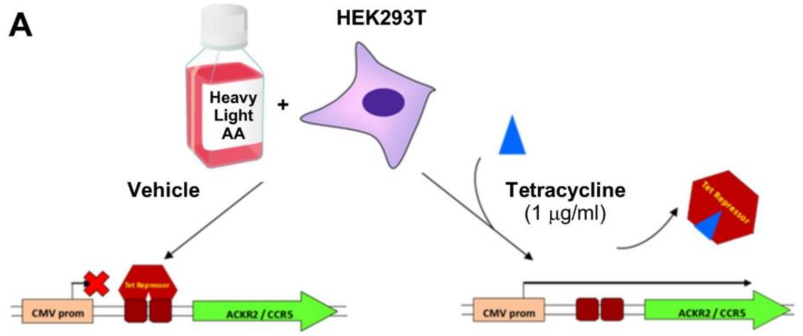

B

	SAMPLE	TET	MEDIUM	STIMULUS
1	Basal (ACKR2 cells)	-	Light	Vehicle
2	Basal (CCR5 cells)	-	Light	Vehicle
3	ACKR2 constitutive	+	Heavy	Vehicle
4	CCR5 constitutive	+	Heavy	Vehicle
5	ACKR2 short-term	+	Light	100 nM CCL3L1, 3’
6	ACKR2 long-term	+	Light	100 nM CCL3L1, 30’
7	CCR5 short-term	+	Light	100 nM CCL3L1, 3’
8	CCR5 long-term	+	Light	100 nM CCL3L1, 30’

C

ANALYSIS	CODE	SAMPLE COMPARISON
ACKR2 constitutive activity	ACKR2_T0	1 vs 3
CCR5 constitutive activity	CCR5_T0	2 vs 4
ACKR2 induced, short-term activity	ACKR2_T3	3 vs 5
ACKR2 induced, long-term activity	ACKR2_T30	3 vs 6
CCR5 induced, short-term activity	CCR5_T3	4 vs 7
CCR5, induced long-term activity	CCR5_T30	4 vs 8

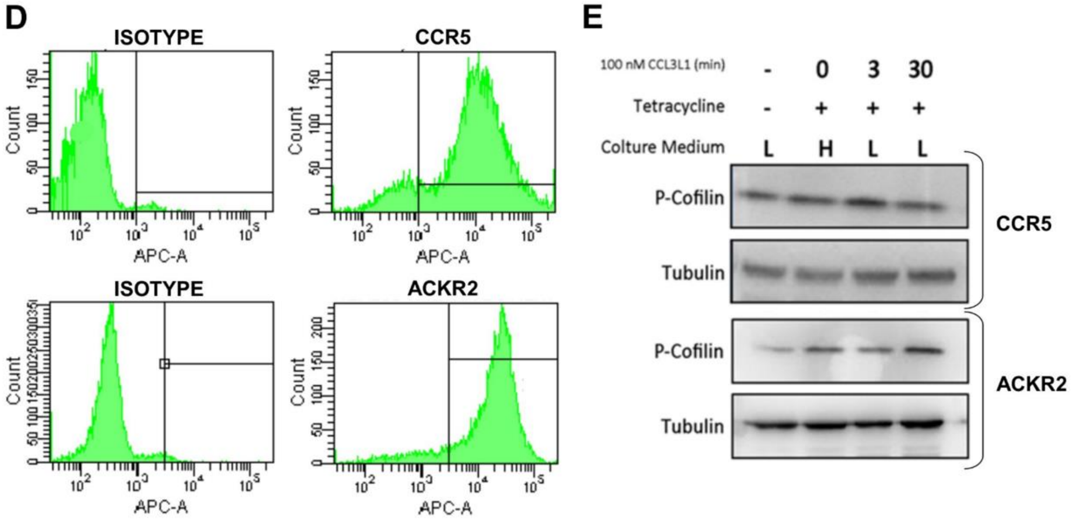

Figure S1. Experimental design and technical controls.

A) Schematic representation of the SILAC procedure. B) Experimental samples generated for SILAC analysis. C) Comparisons used to define constitutive and agonist-induced receptors’ activities. D) Receptor expression after 24 h treatment with 1 ug/ml tetracycline has been evaluated by flow cytometry analysis. One representative cell preparation is shown. (E) Western blotting analysis has been used to evaluate cofilin phosphorylation in tetracycline-treated cells, both in unstimulated conditions and after treatment with 100 nM CCL3L1 at indicated time points. Tubulin was used to normalize protein load. One representative cell preparation is shown.

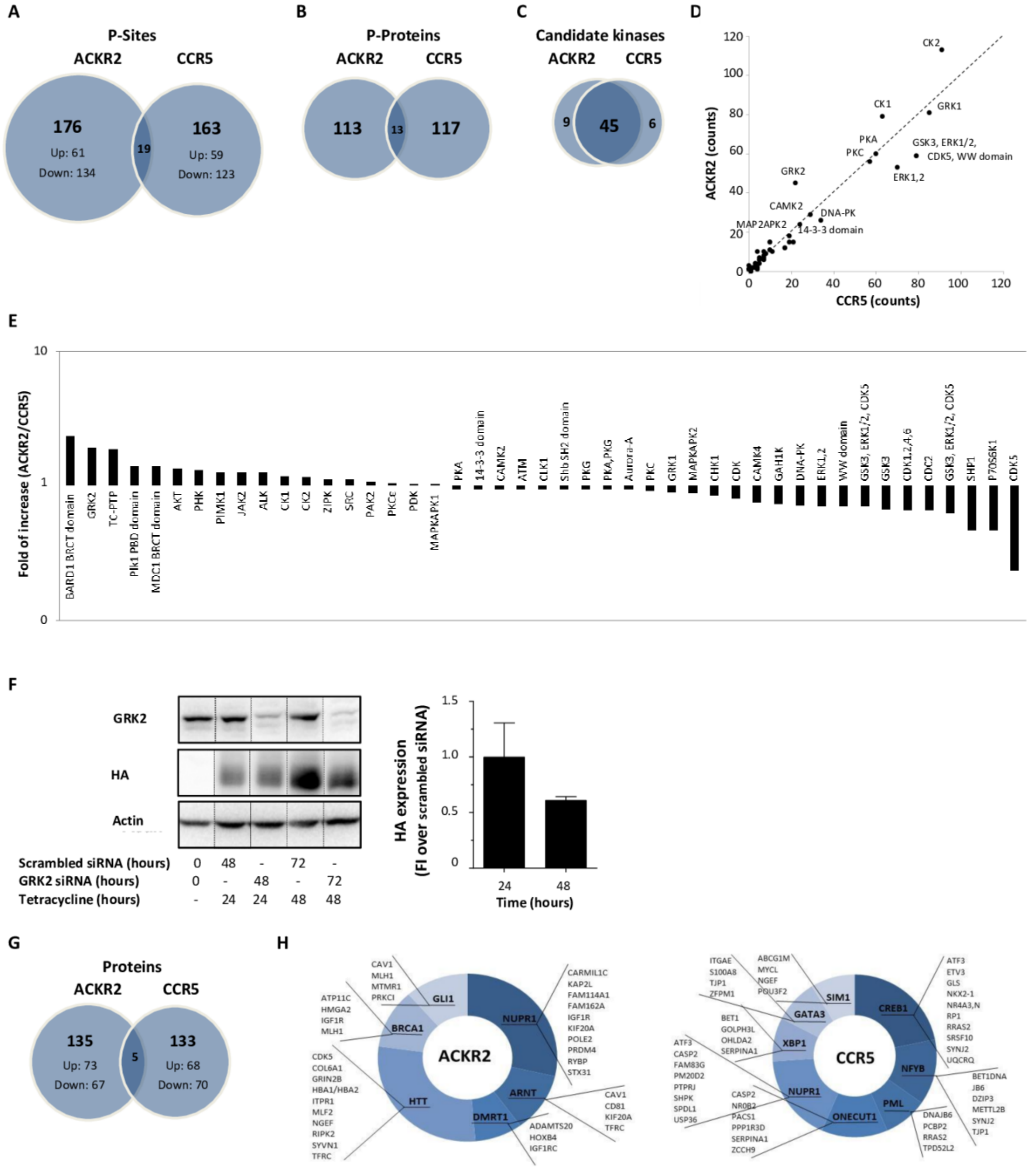

Figure S2. Effect of ACKR2 or CCR5 expression on constitutive phosphoproteome.

A-B) Number of phosphosites (A) and phosphoproteins (B) regulated 24 h after induction of ACKR2 or CCR5 expression in HEK293 T-Rex cells.

C-D) Number of kinases (C) identified by sequence motif analysis in ACKR2- and CCR5-regulated phosphosites, shown in D as absolute counts of related phosphorylation sequence motifs.

E) Ratio of phosphosite percentages associated to kinases activated in ACKR2- and CCR5-expressing cells as identified by phosposite analysis.

F) Effect of GRK2 silencing on constitutive HA-ACKR2 expression in HEK293 T-Rex cells. Blots from one experiment representative of two performed are shown on the left, protein quantification is shown on the right. Results are the means ± SEM of n=2 independent experiments. FI: Fold of increase.

G-H) Number of proteins differentially expressed in HEK293 T-Rex cells 24 h after induction of ACKR2 or CCR5 expression. In panel H, the inner circle reports transcription factors active in

ACKR2- and CCR5-expressing cells based on Ingenuity Pathway Analysis of enriched transcriptional consensus sites, the outer circle reports the respective differentially expressed proteins.

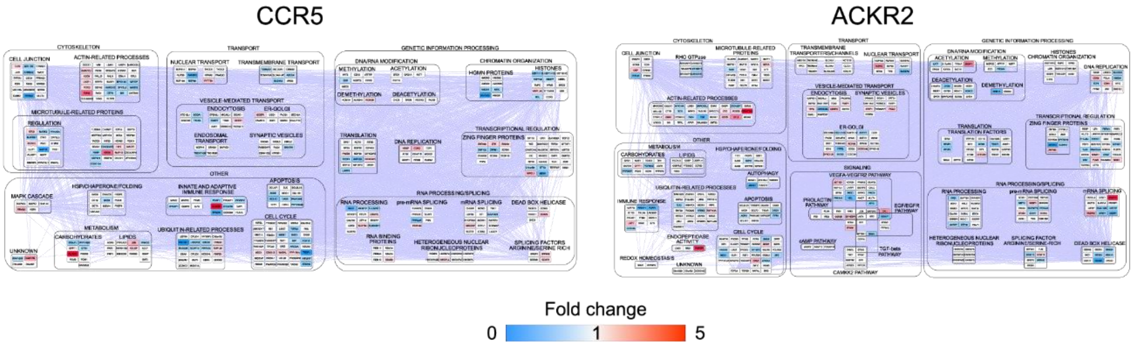

Figure S3. Protein-protein interaction (PPI) network models reconstructed starting from differentially phosphorylated proteins (DPPs) in constitutive CCR5 and ACKR2 phosphoproteome.

Functional modules enriched in HEK293 T-Rex cells constitutive expressing CCR5 and ACKR2. Network models (CCR5: 435 nodes and 4911 edges; ACKR2: 490 nodes and 7151 edges) were reconstructed by STRING Cytoscape's APP. Node color agrees with the fold change of phosphorylated proteins at constitutive phosphoproteome.

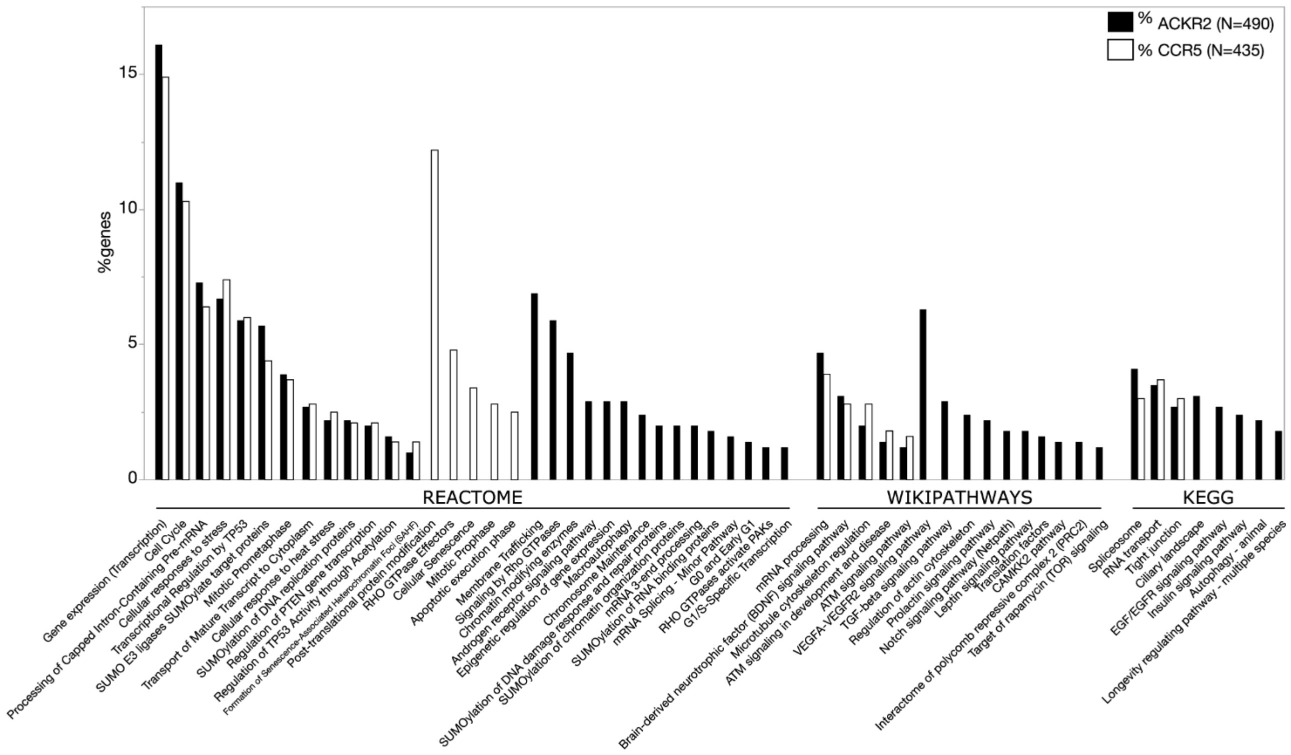

Figure S4. Pathways enriched in protein-protein interaction (PPI) network models reconstructed starting from differentially phosphorylated proteins (DPPs) in CCR5 and ACKR2.

Enriched pathways (FDR<0.05, P value<0.001) extracted by STRING Cytoscape's APP and considering Reactome, WikiPathways and KEGG databases. For each pathway, the percentage of the corresponding identified genes/proteins is reported for both CCR5 (white bars) and ACKR2 (black bars).

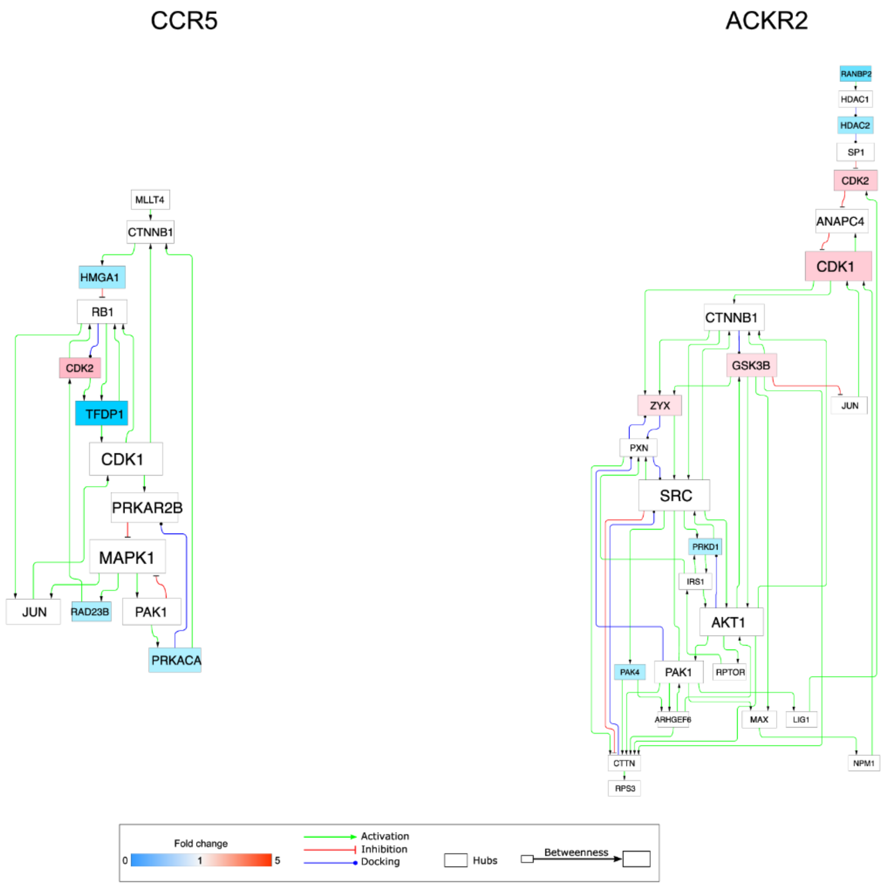

Figure S5. Protein hubs characterizing CCR5 and ACKR2 signaling network models.

Protein hubs selected by betweenness in CCR5 signaling network (80 nodes and 144 edges) and in ACKR2 signaling network (135 nodes and 276 edges). Node color agrees with the fold change of phosphorylated proteins at constitutive phosphoproeome, while node and font size are in agreement with the corresponding betweenness value.

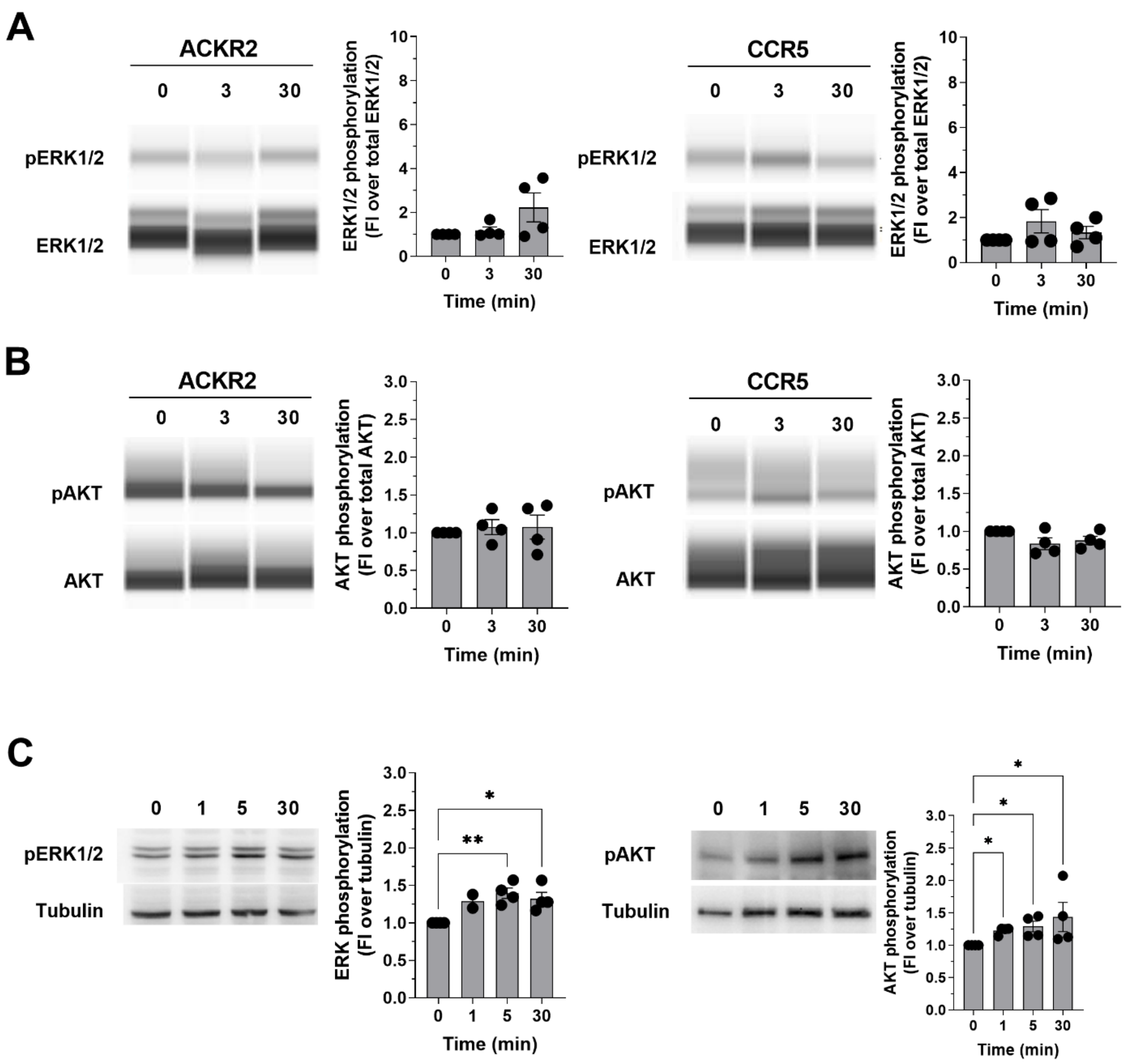

Figure S6. Phosphorylation of ERK1/2 and AKT in ACKR2 and CCR5 cells.

ERK1/2 phosphorylation at T202/Y204 residue and AKT phosphorylation at S473 residue in tetracycline-untreated HEK293 T-Rex cells (WES: A-B) or ACKR2-espressing CHO-K1 cells (Western Blotting: C) following stimulation with 100 nM CCL3L1 at indicated time points. Representative blots are shown on the left, quantification of the relative number of phosphorylated proteins is shown on the right and is indicated as fold increase in phosphorylated proteins abundance compared to that in untreated cells. Data information: statistical analysis by Kruskal-Wallis' multiple comparison test (* $p<0.05$, ** $p<0.01$ for CCL3L1-stimulated cells versus untreated cells). FI: Fold of increase.

SUPPLEMENTARY TABLES

Table S1. CCL3L1-induced phosphoproteome in ACKR2 and CCR5-expressing cells.
A) Absolute counts and percentages of regulated phosphosites and phosphoproteins; absolute counts. B-C) Percentages (B) and lists (C) of regulated protein kinases, phosphatases and GTPase/ATPase; lists of candidate kinases identified by sequence motif analysis of regulated phosphosites at time T3 (D) and T30 (E) of stimulation with 100 nM CCL3L1, with their absolute counts, percentages and fold of increase calculated as ACKR2 percentage over CCR5 percentage.

Table S2. Constitutive phosphoproteome profile in ACKR2- and CCR5-expressing cells.

Absolute counts and percentages of regulated phosphosites and phosphoproteins (A), and candidate kinases identified by sequence motif analysis of regulated phosphosites with their absolute counts, percentage, and fold of increase calculated as ACKR2 percentage over CCR5 percentage (B).

Table S3. Proteins differentially expressed in ACKR2- and CCR5-expressing cells.

A) Absolute counts and percentages of protein with differential expression in ACKR2- or CCR5-expressing cells as compared to uninduced HEK293 T-Rex cells. (B) GeoMean of proteins: upregulated in both ACKR2 and CCR5, down-regulated in both ACKR2 and CCR5, up-regulated in ACKR2 and down-regulated in CCR5, up-regulated only in ACKR2, down-regulated only in ACKR2, up-regulated only in CCR5, down-regulated only in CCR5.

Table S4. Enriched pathways in ACKR2- and CCR5-expressing cells.

Enriched pathways (FDR<0.05, P value<0.001) extracted by STRING Cytoscape's APP and considering Reactome, WikiPathways and KEGG databases. For each pathway, the number and percentage of the corresponding identified genes/proteins is reported for both CCR5 (N=435) and ACKR2 (N=490).

Table S5. Protein hubs in ACKR2- and CCR5-expressing cells.

Protein hubs selected by betweenness in (A) CCR5 signaling network (80 nodes and 144 edges) and in (B) ACKR2 signaling network (135 nodes and 276 edges). For each hub, the Betweenness, Bridging, Centroid, Closeness, Eccentricity, EigenVector, Radiality, Stress, InDegree and OutDegree values are shown. In addition, the fold change at T3, T30 and constitutive (T0) is reported, too.
