# Supplementary figures and images for: Phosphoproteomic mapping of CCR5 and ACKR2 signaling properties
Source: Front Mol Biosci. 2022 Nov 22;9:1060555. doi: 10.3389/fmolb.2022.1060555 (PMC9723398; doi:10.3389/fmolb.2022.1060555)

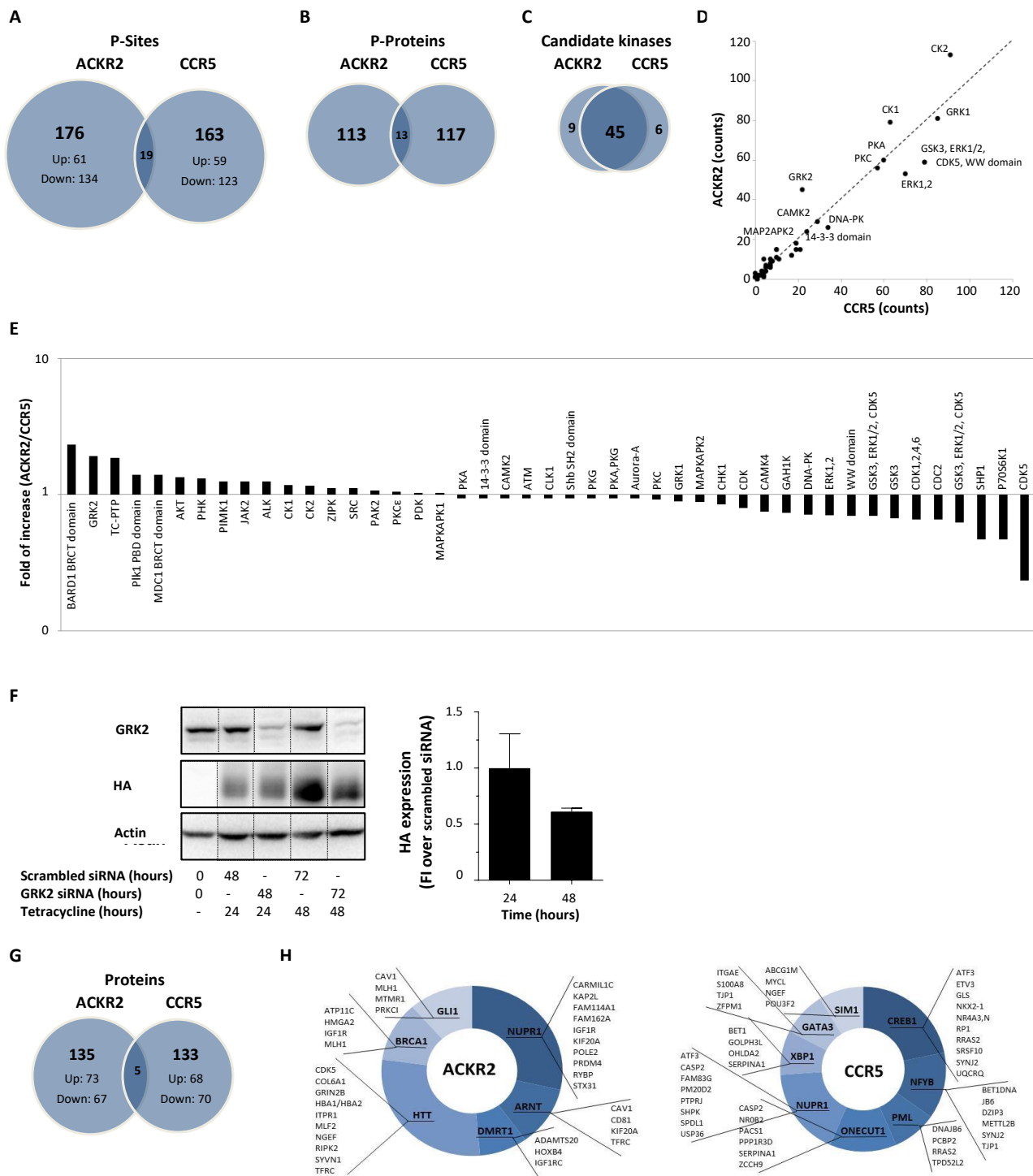

Supplement: Supplementary file 1 [file DataSheet1.zip › Data Sheet ZIP folder/Figure S2.pdf]

## CCR5

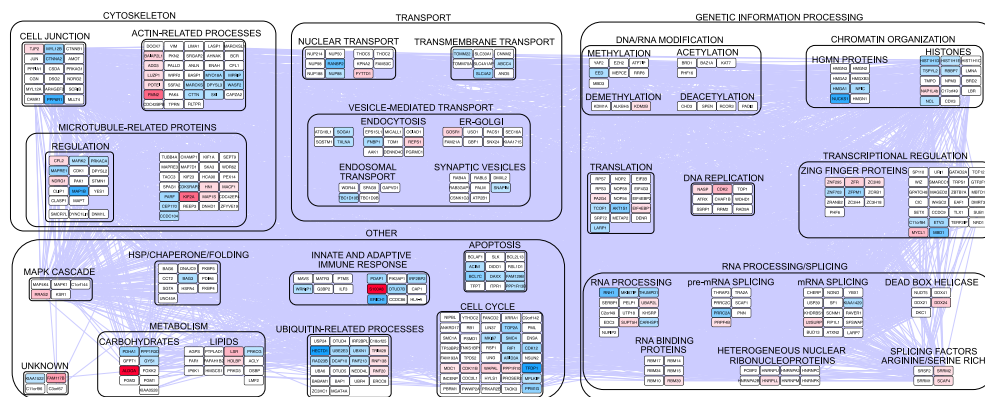

## ACKR2

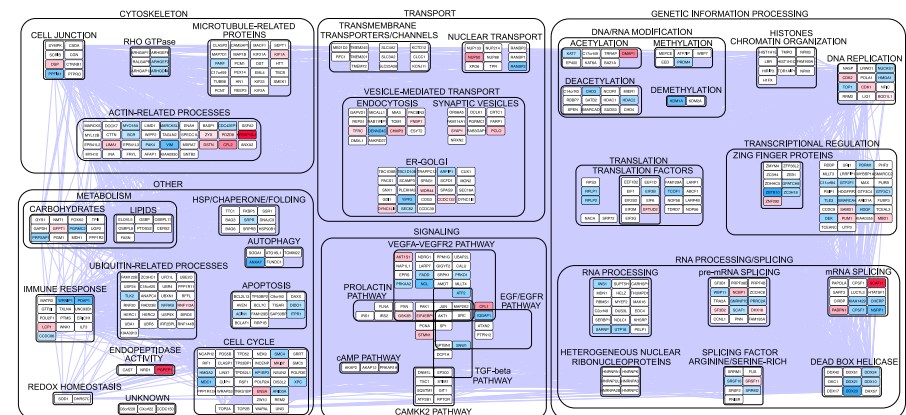

Fold change

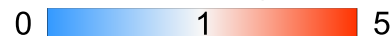

Supplement: Supplementary file 1 [file DataSheet1.zip › Data Sheet ZIP folder/Figure S3.pdf]

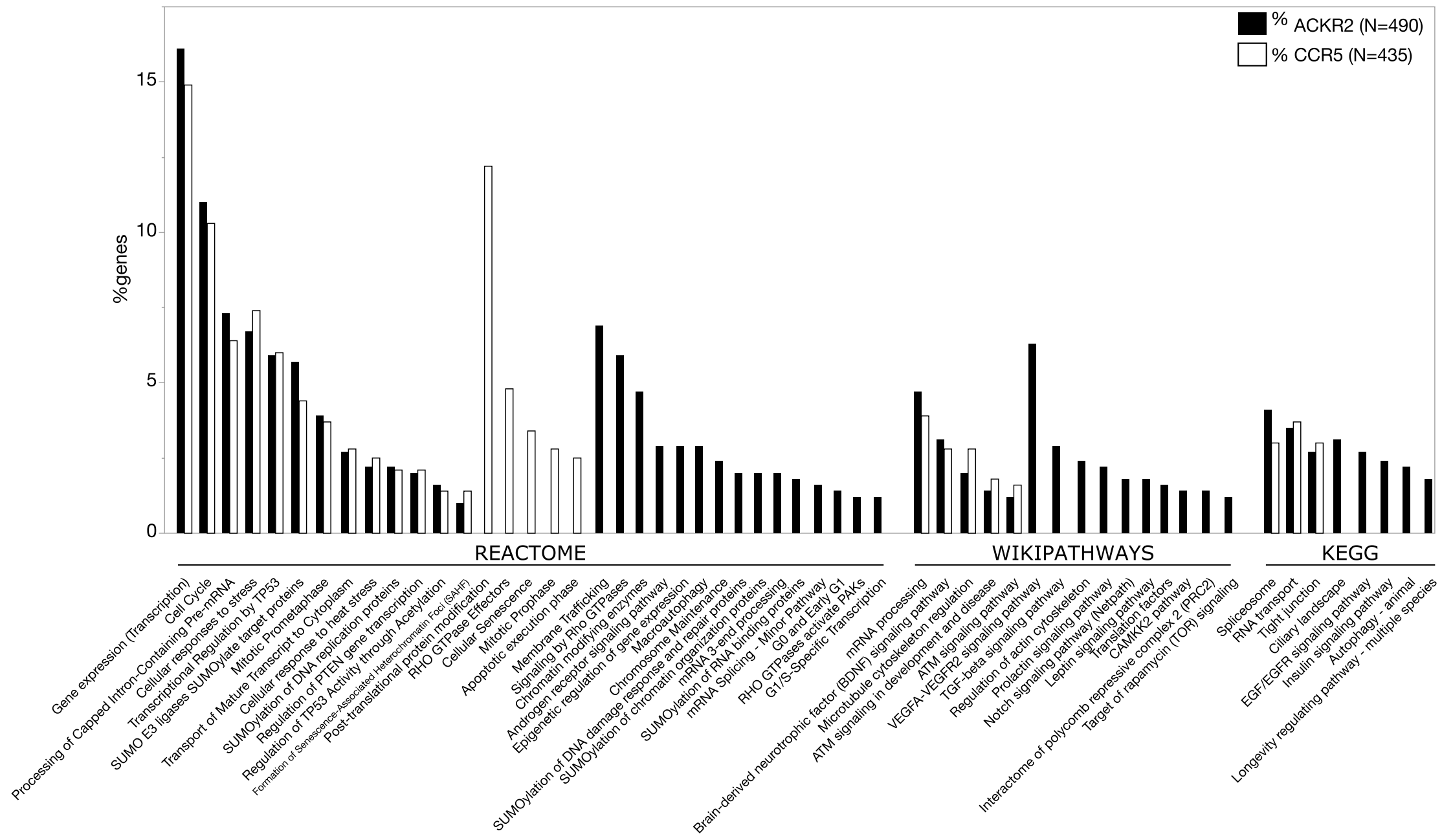

Supplement: Supplementary file 1 [file DataSheet1.zip › Data Sheet ZIP folder/Figure S4.pdf]

# CCR5

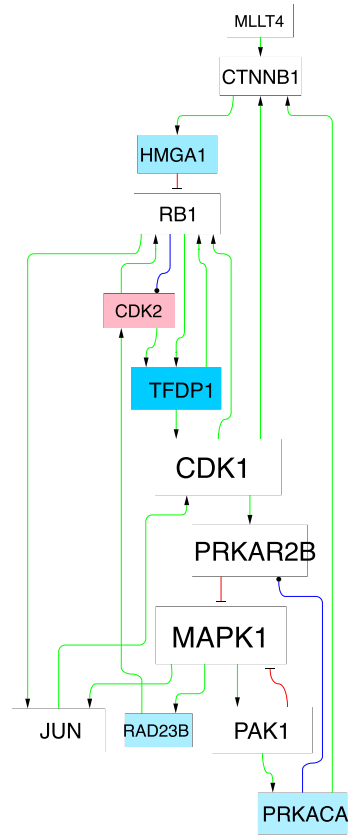

# ACKR2

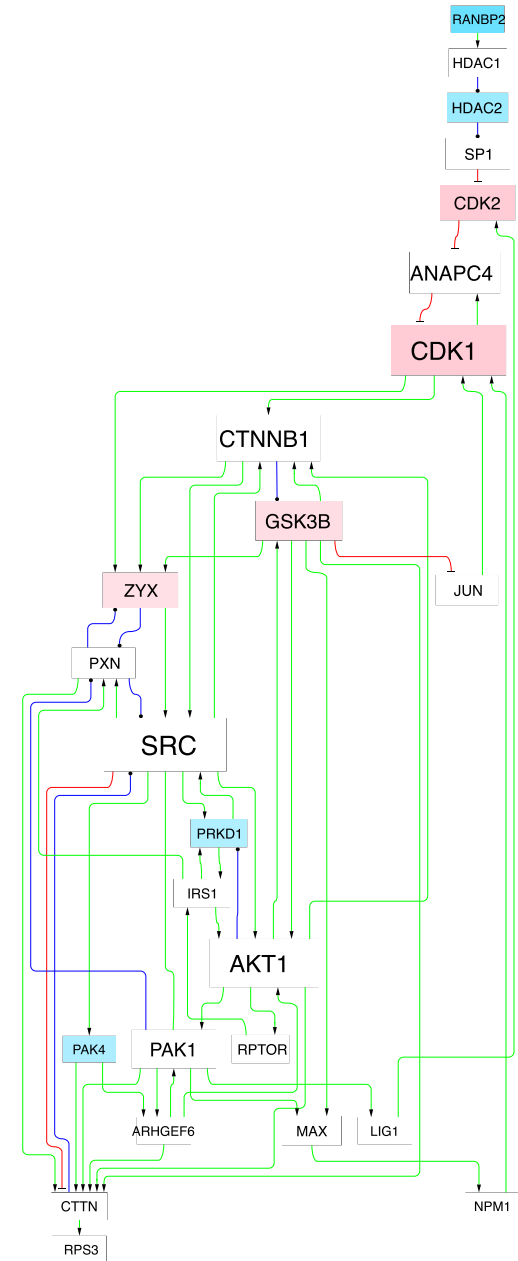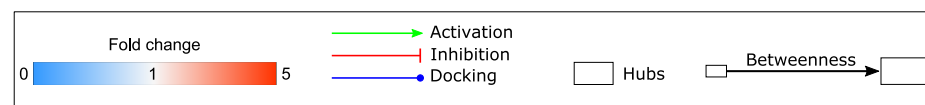

Supplement: Supplementary file 1 [file DataSheet1.zip › Data Sheet ZIP folder/Figure S5.pdf]

**A**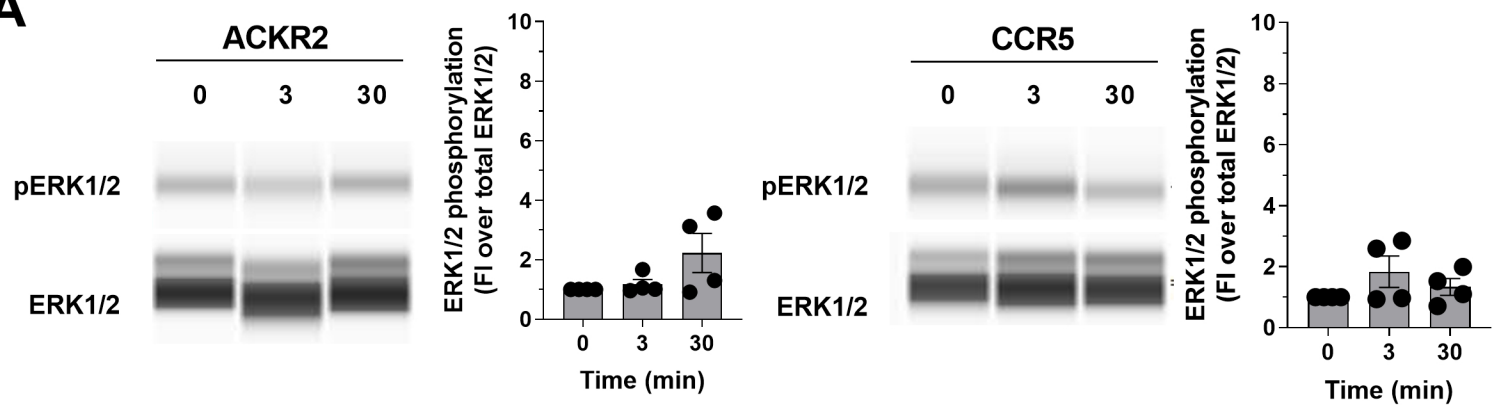**B**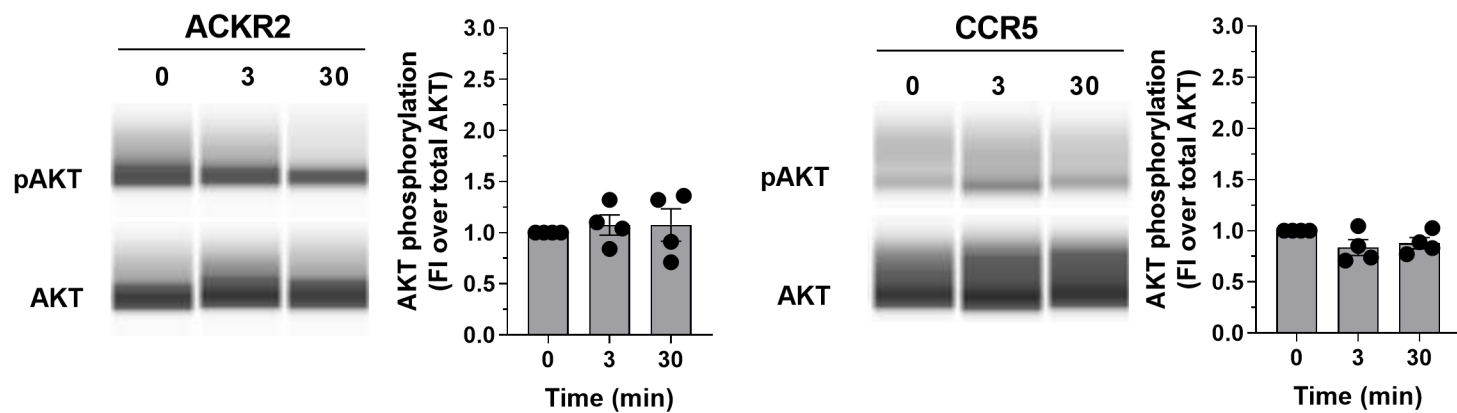**C**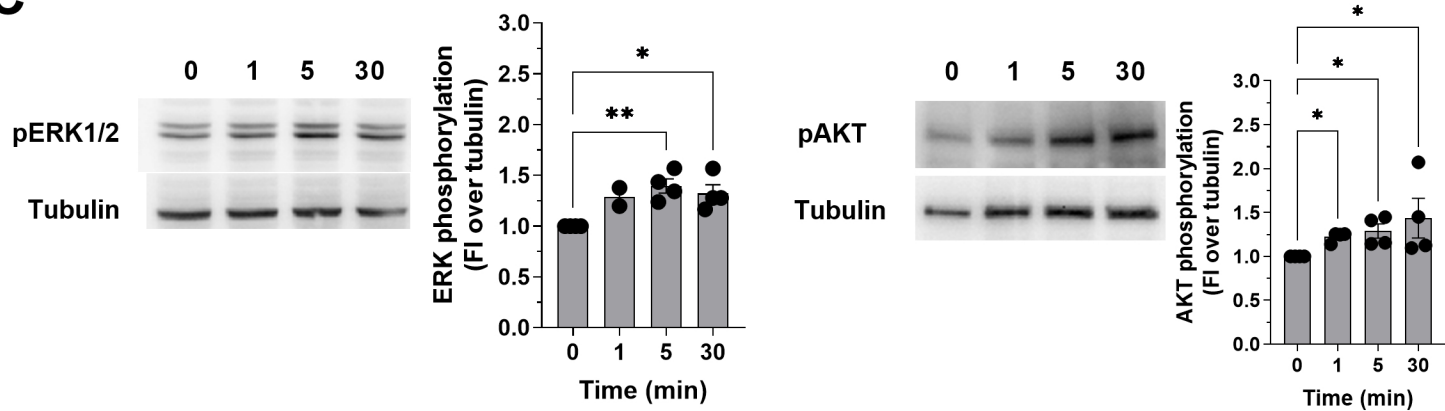

Supplement: Supplementary file 1 [file DataSheet1.zip › Data Sheet ZIP folder/Figure S6.pdf]
